# DNA-Based Environmental Remediation: Functional Principles, Material Platforms, and Future Perspectives

**DOI:** 10.3390/ma19173707

**Published:** 2026-08-31

**Authors:** Minhyuk Lee, Hamin Park, Sungjee Kim, Nokyoung Park

**Affiliations:** 1 Department of Chemistry, Pohang University of Science and Technology, Pohang 37673, Republic of Korea; 2 Department of Chemistry, Myongji University, 116 Myongji-ro, Yongin-si 17058, Republic of Korea

**Keywords:** DNA-based environmental remediation, DNA nanotechnology, pollutant removal, aptamer, programmable DNA materials

## Abstract

With advancements in DNA nanotechnology, the role of DNA has expanded far beyond its traditional function as genetic material, and it is now increasingly utilized as a programmable material distinguished by specific molecular recognition and controllable self-assembly. As contemporary environmental pollutants become increasingly complex, conventional remediation technologies often face critical limitations due to their poor selectivity and low adaptability. Consequently, DNA nanotechnology presents a promising alternative for intelligent remediation. Specifically, functional nanostructures such as aptamers, DNAzymes, hydrogels, and hybrid nanocomposites serve as innovative platforms for the highly selective sequestration, degradation, and isolation of diverse contaminants. This review summarizes the fundamental properties of DNA relevant to environmental remediation, including molecular recognition and catalytic activity, while also exploring underutilized functions with potential for future remediation applications, such as enzyme-free amplification and stimulus-responsive structural transitions. We further discuss diverse DNA-based material platforms and systematically examine reported remediation strategies across major classes of environmental pollutants, with particular emphasis on aqueous systems, which currently represent the primary context of experimentally demonstrated DNA-based pollutant removal and degradation. Beyond these demonstrated strategies, we also explore emerging DNA-based concepts with potential for future remediation applications, particularly through the expansion of DNA functionalities, integration with advanced material platforms, and extension to new classes of target pollutants. Finally, we discuss the key challenges associated with practical environmental translation, providing an integrated perspective on both the current landscape and future development of DNA-based remediation technologies.

## 1. Introduction

Deoxyribonucleic acid (DNA) is a biopolymer composed of four nucleotide bases, adenine (A), thymine (T), cytosine (C), and guanine (G), and it stores genetic information in its nucleotide sequence. The most inherently unique characteristic of DNA lies in the sequence-specific interactions of these four bases, known as Watson–Crick base pairing, which enables not only precise structural control at the nanometer scale but also the sophisticated recognition and capture of targets ranging from biomacromolecules to small organic molecules [1,2,3,4]. Driven by these excellent properties and the rapid advancements in nanotechnology, the versatility and utility of DNA as a highly versatile functional material beyond its traditional biological role as a carrier of genetic information have become increasingly recognized. The programmability of DNA has enabled a wide range of applications in biomedicine, molecular computing, and information storage [2,4,5,6,7,8,9]. In addition, although widespread practical implementation remains limited, functional DNA technologies such as aptamers, DNAzymes, and isothermal amplification of nucleic acids have been widely studied as platforms for environmental monitoring and pollutant detection based on their high selectivity and sensitivity [10,11,12]. More recently, DNA-based materials are attracting attention as promising next-generation platforms not only for environmental monitoring but also in the field of environmental remediation for the capture, degradation, and removal of pollutants due to their precise molecular recognition capabilities, adaptability, and stimulus responsiveness [13,14,15,16].

This growing interest stems from the fact that contemporary environmental pollution has become increasingly diverse and complex, exposing critical limitations in the real-world application of conventional remediation technologies. Today, rapid population growth, industrialization, and urbanization have led to increasingly complex environmental challenges characterized by the widespread coexistence of diverse contaminants, while advances in analytical technologies and the continued identification of emerging contaminants have further increased the performance requirements for effective remediation and have expanded the range of recognized environmental pollutants, including heavy metals, organic pollutants, pharmaceutical residues, pesticides, and pathogenic microorganisms [17,18]. Owing to their persistence, toxicity, and potential for bioaccumulation, these contaminants pose serious risks to ecosystems, water resources, and human health. Consequently, the establishment of efficient and reliable technologies for intelligent environmental management and remediation has emerged as a critical global priority. However, conventional environmental systems typically suffer from decoupled recognition and remediation processes, often requiring multistep external operations and manual intervention to address complex environmental challenges [16]. Moreover, maintaining high selectivity and performance under conditions where diverse contaminants and interfering substances coexist remains a persistent challenge in real-world environmental matrices [18,19]. Therefore, DNA-based materials have emerged as particularly attractive candidates for environmental applications owing to their ability to integrate molecular recognition, stimuli-triggered operation, and contaminant removal within a single programmable platform [10,16,20]. In addition, DNA-based materials generally exhibit low intrinsic toxicity and can be degraded into naturally occurring nucleotides, potentially reducing concerns regarding secondary environmental contamination.

**Figure 1:**
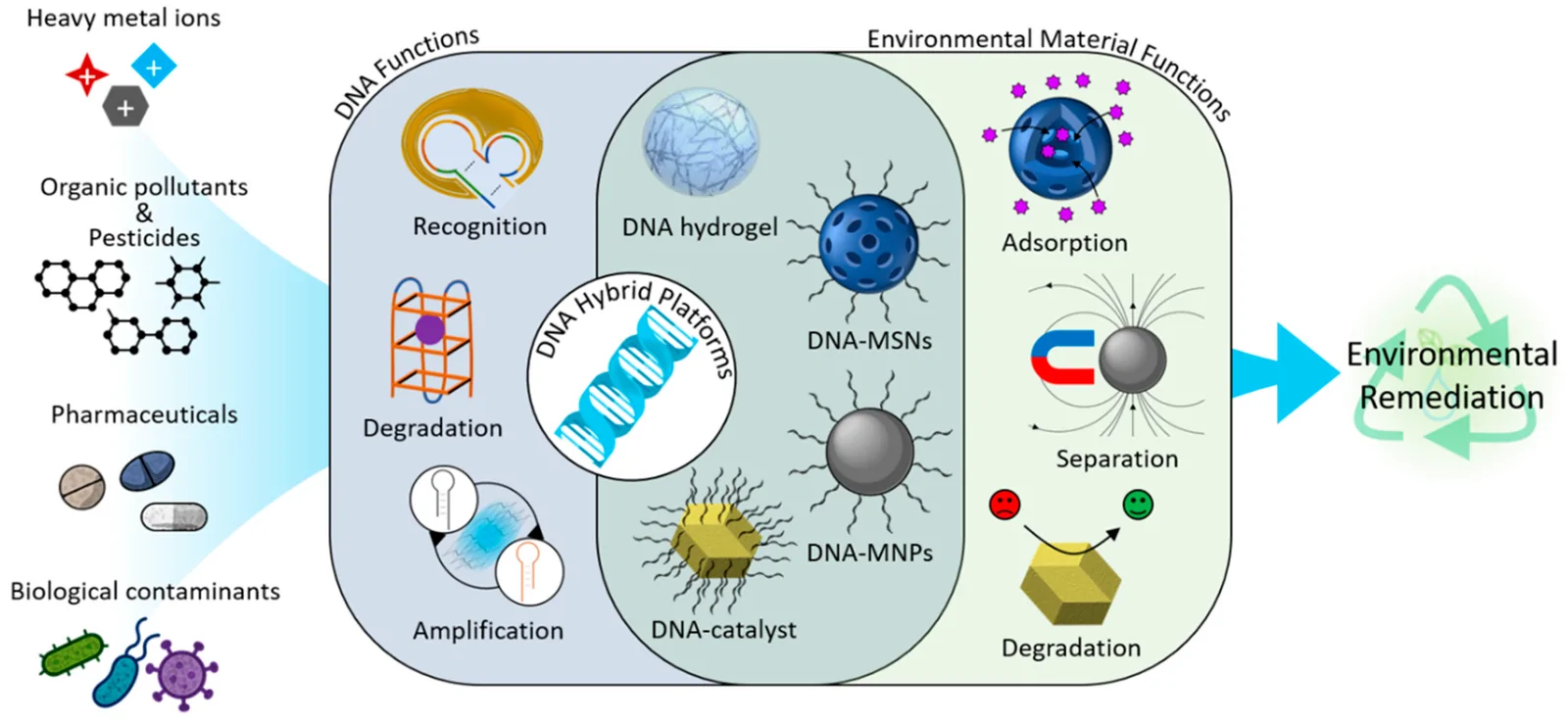
Schematic illustration of DNA-based materials for environmental remediation. Environmental pollutants, including heavy metals, organic contaminants, pesticides, pharmaceuticals, and biological contaminants, are recognized and processed by DNA-based materials. Through programmable structural architectures such as DNA hydrogels, MSNPs, MNPs, and DNA-catalysts and diverse functional capabilities such as target recognition, catalytic degradation, and structural amplification, DNA-based materials enable selective pollutant remediation. The arrows indicate the overall flow from environmental pollutants through remediation systems to their remediation.

Conventional remediation materials, including activated carbon, zeolites, and metal–organic frameworks (MOFs), possess distinct and well-established strengths in adsorption performance, physicochemical stability, structural or surface tunability, and practical applicability [21,22,23]. However, these materials also present material- and application-specific limitations related to factors such as selectivity, regeneration, recovery, and processability [21,22,23]. DNA-based materials provide a different set of advantages arising from their intrinsic molecular interactions and structural and functional programmability, although their standalone use may be constrained by environmental stability and the cost and scalability associated with large-scale nucleic acid production [13,24,25]. Importantly, DNA and conventional remediation materials can be integrated into complementary hybrid systems that combine their respective strengths while mitigating some of their individual limitations. In such systems, established remediation materials can provide adsorption, catalytic, structural, and practical advantages, whereas DNA can introduce additional functions such as molecular recognition, catalysis, structural programmability, and stimuli-responsive behavior [13,24,25]. Beyond this functional complementarity, the practical limitations of DNA-based materials provide an additional rationale for such integration. Incorporating functional DNA into established remediation materials may improve DNA stability and reduce reliance on DNA as the bulk material, potentially alleviating some of the cost and scalability constraints associated with DNA-only systems. Specifically, this platform enables the easy incorporation of aptamers, short single-stranded nucleic acids selected through screening processes for highly specific binding to target molecules [24,25]. Aptamers can be integrated with heterogeneous nanomaterials, such as carbon nanotubes, graphene, gold nanoparticles, magnetic nanoparticles (MNPs), mesoporous silica nanoparticles (MSNPs), and metal–organic frameworks, to construct hybrid nanocomposites that combine the exceptional molecular recognition capability of aptamers with the high surface area, adsorption capacity, and catalytic properties of nanomaterials, thereby enabling selective pollutant capture and enhanced remediation performance [24,25]. Furthermore, through integration with enzyme-free isothermal nucleic acid amplification cascades such as the hybridization chain reaction (HCR), this system can autonomously assemble into 3D networked DNA hydrogels upon target recognition, providing an excellent macrostructural matrix for the high-capacity sequestration and isolation of specific pollutants [10,26,27]. This multifunctional versatility shifts environmental management from conventional passive treatments to intelligent, interactive processes, thereby serving as an innovative platform capable of simultaneously achieving the selective recognition, targeted capture, and catalytic degradation of diverse contaminants within complex ecosystems (Figure 1) [13,16].

In this review, we provide an integrated overview of recent advances in programmable DNA nanotechnology for environmental remediation, encompassing both experimentally demonstrated remediation strategies and emerging concepts with potential for future remediation applications. This review primarily considers studies directly related to DNA-based environmental remediation, covering a broad range of DNA functions, material platforms, target pollutants, and remediation strategies. The available experimental evidence for DNA-based environmental remediation is currently concentrated predominantly on aqueous systems, including water and wastewater treatment. Accordingly, particular emphasis is placed on remediation strategies demonstrated in or proposed for aqueous environments, while their potential extension to other environmental matrices is also considered. We first discuss the fundamental molecular functions of DNA, including aptamer-mediated recognition and DNAzyme-mediated catalysis, as well as enzyme-free isothermal amplification, which has been primarily explored in sensing and monitoring but remains largely unexplored for environmental remediation. We then review the major material platforms and structural design strategies relevant to DNA-based remediation, including hydrogels, porous and magnetic nanomaterials, membranes, and catalytic systems. Subsequently, we systematically examine the application of these DNA-based platforms to major classes of environmental pollutants, including heavy metals, organic dyes, biotoxins, biological contaminants, and emerging contaminants such as pharmaceuticals and per- and polyfluoroalkyl substances (PFASs), with a primary focus on experimentally demonstrated pollutant removal and degradation. Finally, we explore future opportunities for expanding DNA-based remediation by broadening DNA functionalities, integrating DNA with advanced material platforms, and extending its application to new classes of environmental pollutants while also considering the practical challenges associated with their translation to real-world environmental applications. Through this perspective, we aim to provide a framework for understanding the current landscape of DNA-based environmental remediation and identifying opportunities to expand its molecular functions, material platforms, and target pollutants to future remediation technologies.

## 2. Molecular Functions of DNA for Environmental Remediation

**Figure 2:**
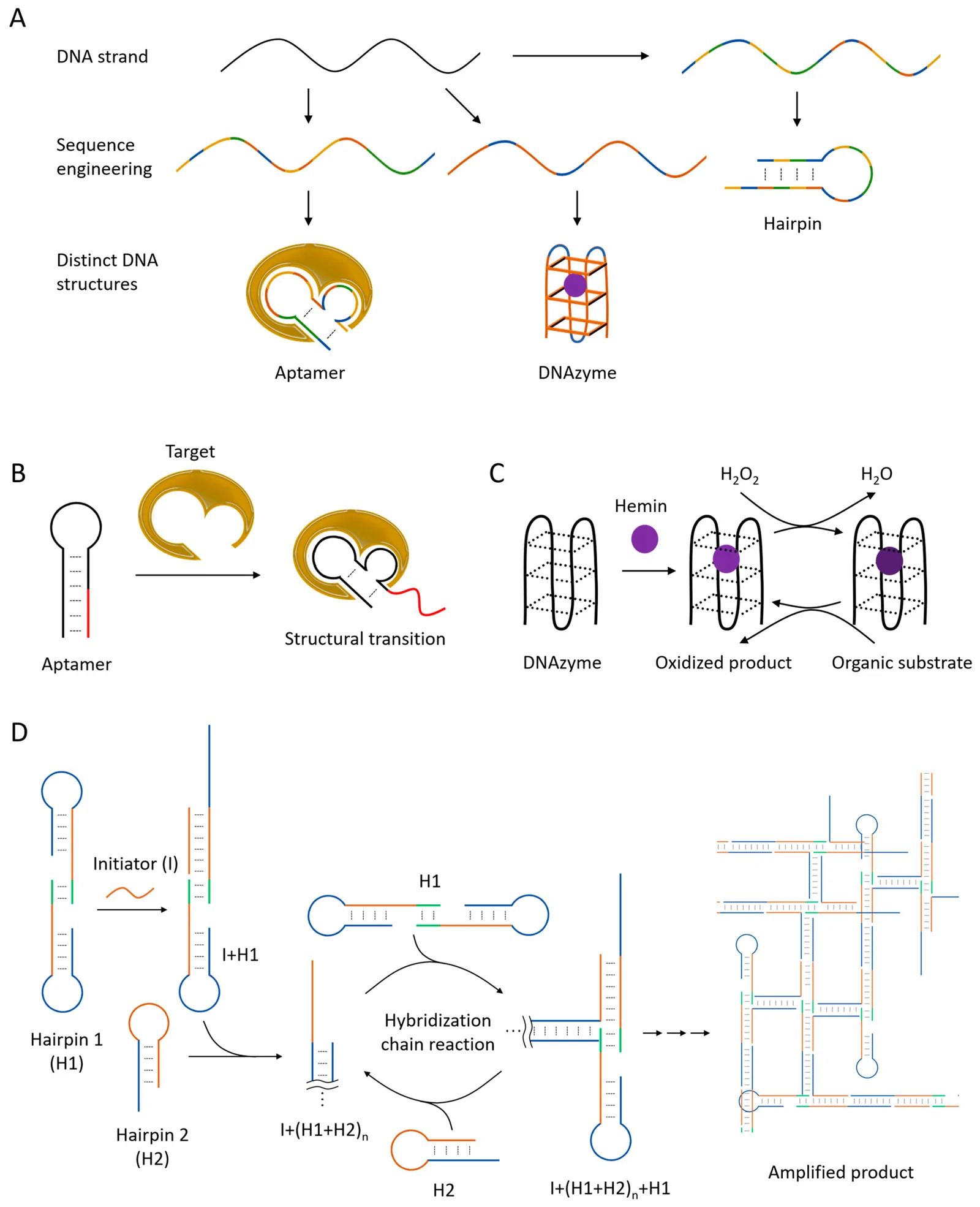
Representative functional mechanisms enabled by DNA sequence programmability. (**A**) Sequence programmability of DNA. Distinct nucleotide sequences undergo sequence-dependent folding into diverse functional structures, including aptamers, DNAzymes, and hairpin motifs. Different colors represent different nucleotide bases, and the arrows indicate the diversification of DNA functions arising from sequence-dependent folding. (**B**) Aptamer-mediated target recognition through sequence-dependent folding and conformational switching. The red region represents a sequence exposed following target-induced conformational change. (**C**) Catalytic transformation of organic substrates by G-quadruplex/hemin DNAzymes. (**D**) Enzyme-free amplification and structural assembly through Clamped-HCR. matching colors indicate complementary DNA sequences, and the consecutive arrows indicate multiple successive reaction steps. In the context of environmental remediation, DNAzyme-mediated catalytic transformation (**C**) has been experimentally demonstrated, whereas the downstream use of target-induced conformational switching (**B**) and enzyme-free amplification (**D**) represents prospective functionality.

To critically evaluate the potential of DNA-based environmental remediation systems, a fundamental understanding of the intrinsic molecular functionalities of DNA is essential. These functionalities can be broadly categorized into (1) target-specific molecular recognition, (2) catalytic transformation and degradation, and (3) enzyme-free self-assembly and signal amplification. In fact, all of these mechanisms originate fundamentally from the structural flexibility of single-stranded DNA and the programmability of nucleotide sequences (Figure 2A). These allow DNA to exert diverse and unique functions through unique folding and hybridization behaviors. Despite this excellent potential, DNA-based environmental applications have so far been concentrated in the monitoring field, and only recently has their scope expanded to environmental remediation [10,12,13,28]. Although some of these functionalities have not yet been fully exploited for environmental remediation, understanding their underlying mechanisms provides opportunities for the rational design of more advanced DNA-based remediation platforms. In this section, we comprehensively review the working principles and rational design strategies underlying these three core molecular functionalities of DNA nanotechnology, providing a conceptual framework for understanding how they are implemented in the diverse DNA-based remediation platforms discussed in the following section and how they may be further exploited in next-generation systems.

### 2.1. Target-Specific Molecular Recognition

In complex aquatic environments where numerous dissolved species coexist, the ability to selectively distinguish target contaminants from competing substances is essential for practical remediation. Among the various excellent functions of DNA, molecular recognition is one of the most powerful capabilities enabling selective pollutant capture and isolation. Aptamers refer to short single-stranded DNA that has high affinity and selectivity for specific targets through sequence-dependent folding and molecular interactions. Aptamers are typically 20–100 nucleotides in length and are obtained through the systematic evolution of ligands by the exponential enrichment (SELEX) process [4,24,25]. The SELEX process starts with a massive synthetic library containing up to 10^16^ highly diverse and randomized oligonucleotide sequences. Subsequently, through repetitive binding–separation–amplification cycles, sequences that bind strongly to the target are selectively enriched, ultimately yielding aptamers with high affinity and selectivity. Through this iterative screening process, aptamers with high affinity and selectivity have been identified for diverse environmental contaminants, including heavy metal ions such as Hg^2+^, Pb^2+^, and Cd^2+^; organic pollutants such as pesticides, antibiotics, and endocrine disruptors; and biological contaminants such as pathogenic microorganisms [24,25,29]. More recently, aptamers capable of recognizing PFASs, an important class of emerging contaminants receiving increasing environmental attention, have also been reported [30]. Notably, an ssDNA aptamer selected for perfluorooctanoic acid (PFOA), a member of the PFAS family, exhibited different binding affinities for structurally related PFASs, indicating that even contaminants within the same chemical class can interact differently with a given aptamer depending on their molecular characteristics [30]. In this study, fluorocarbon chain length was identified as an important factor influencing aptamer binding [30]. These findings highlight that the molecular characteristics of a target can influence aptamer–target interactions. More generally, although there are no universal criteria for predicting whether a high-affinity aptamer can be obtained for a particular contaminant, small molecules with limited structural complexity and few potential interaction sites can be more challenging targets for aptamer selection [31]. Nevertheless, the demonstrated diversity of aptamer targets provides a broad basis for their application in DNA-based environmental remediation strategies, although the achievable binding affinity can vary depending on the molecular characteristics of the target.

Recognition between aptamers and targets is mediated by various non-covalent interactions, including hydrogen bonding, electrostatic interactions, van der Waals forces, hydrophobic effects, and π-π stacking [32,33]. These aptamer–target interactions can induce structural changes that stabilize the target binding state and, in some systems, serve as molecular triggers for subsequent functional responses, such as target-responsive cargo release. Furthermore, such target-induced conformational changes could be coupled with downstream molecular processes, including catalytic DNAzyme activation, strand displacement reactions, HCR, and catalytic hairpin assembly (CHA), offering opportunities to expand the functional capabilities of DNA-based remediation systems (Figure 2B). Thus, aptamers not only enable the selective capture and removal of specific contaminants through molecular recognition but could also function as molecular switches that couple target recognition with downstream functional processes, potentially enabling integrated systems in which contaminant recognition directly initiates a remediation response [4,11]. Another key advantage of aptamers is the ability to customize selectivity during the SELEX process. By incorporating competing ions or structurally similar compounds into the selection process, aptamers can be evolved to preferentially recognize target contaminants while minimizing non-specific interactions [34,35]. This target specificity is particularly important in modern environmental systems where target contaminants coexist with chemically similar substances. Finally, aptamers can be easily integrated with various existing environmental remediation materials, enabling more precise and selective pollutant capture by introducing target-specific molecular recognition into established material platforms [13,15,36,37]. Nevertheless, an important consideration is that the high affinity and selectivity of aptamers are not necessarily invariant under different environmental conditions. Because aptamers are generally selected and characterized under defined experimental conditions, variations in solution chemistry and environmental matrices can alter aptamer folding and target recognition such that binding performance measured under optimized conditions may not directly translate to complex environmental samples [24,25]. In summary, aptamers enable highly selective recognition of target contaminants even in chemically complex environmental matrices and can be utilized as functional modules to impart molecular selectivity to existing remediation materials. Furthermore, their target-induced conformational changes offer the potential to couple molecular recognition with downstream functional processes, providing opportunities for the development of integrated DNA-based systems that combine selective contaminant recognition with additional remediation functions.

### 2.2. Catalytic Transformation and Degradation

Selective recognition and capture can effectively isolate target contaminants from complex environmental matrices. However, complete remediation often requires their transformation into less toxic or environmentally benign products, particularly when pollutants remain chemically present for a long time after capture. Therefore, interest in catalytic strategies capable of converting pollutants into less harmful substances is gradually increasing [38]. Protein enzyme catalysts have long been regarded as one of the most ideal strategies for environmental remediation due to their high substrate specificity, excellent catalytic efficiency, and ability to accelerate reactions under mild conditions [39,40]. However, protein enzymes face challenges in application to real-world environmental systems due to limited stability, high production costs, and complex manufacturing and storage processes. As an alternative to overcome these limitations, DNA-based catalysts are emerging, offering high chemical stability, easy synthesis, excellent programmability, and high compatibility with various DNA-based structures [40,41,42,43]. Interestingly, DNA sequence-dependent folding can not only enable selective target recognition but also generate catalytically active structures that bind to specific cofactors such as metal ions or small molecules to facilitate chemical transformation. Single-stranded DNA molecules with such catalytic activity, known as DNAzymes, are obtained through in vitro selection processes like aptamers, and numerous DNAzymes have been reported to catalyze reactions such as RNA phosphodiester bond cleavage, linkage, phosphorylation, and porphyrin-mediated oxidation [41,44]. Among these, RNA-cleaving DNAzymes (RCDs) and G-quadruplex-based peroxidase-mimicking DNAzymes (PMDs) are of particular interest for environmental monitoring and remediation because of their distinct catalytic functions. RCDs have been widely explored for the selective detection and monitoring of environmental contaminants, particularly metal ions, whereas PMDs can directly catalyze oxidation reactions for pollutant degradation. Importantly, the target-dependent cleavage of and accompanying structural changes in RCDs may also provide opportunities for their future use as molecular switches that trigger downstream remediation processes.

This remarkable ability to endow DNA with catalytic functionality stems from the capacity of specific nucleotide sequences to fold into well-defined three-dimensional structures, precisely spatializing substrates and cofactors in a manner similar to the active sites of natural protein enzymes [41,44]. RCDs are typically activated by specific divalent metal ions, including Mg^2+^, Pb^2+^, Cu^2+^, UO_2_^2+^, and Hg^2+^, which serve as catalytic cofactors by promoting the formation of catalytically competent conformations and facilitating phosphodiester bond cleavage within RNA or RNA-containing substrates [44]. In particular, since some of these metal ions are environmentally important pollutants, RCDs provide a unique mechanism in which contaminant recognition can directly induce catalytic cleavage and subsequent structural changes [10,12]. Although RCD-based switching mechanisms have been extensively exploited in sensing and functional nanodevices, their application to environmental remediation remains largely unexplored [40,45]. Nevertheless, target-induced RCD cleavage could potentially be utilized to trigger DNA structural rearrangement, hydrogel degradation, or the controlled release of functional components, offering opportunities for dynamically regulated DNA-based environmental remediation systems. In contrast to RCDs, PMDs catalyze redox reactions rather than nucleic acid cleavage. PMDs are typically constructed by the non-covalent assembly of guanine-rich DNA sequences into G-quadruplex (G4) structures, followed by the incorporation of hemin as a catalytic cofactor [38,40]. These G-quadruplex/hemin complexes catalyze oxidation reactions capable of degrading organic contaminants, dyes, and phenolic compounds in the presence of hydrogen peroxide (Figure 2C) [40]. However, the poor oxidative stability of hemin may limit the practical application of these catalytic systems by shortening their catalytic lifetime under H_2_O_2_-dependent reaction conditions [40]. Collectively, RCDs and PMDs both hold potential for environmental remediation, but their current levels of experimental validation differ substantially. While PMDs have been directly applied to oxidative pollutant degradation, the use of RCDs as molecular switches to trigger downstream remediation processes has not yet been experimentally demonstrated. Nevertheless, their distinct functions may offer complementary opportunities for future DNA-based remediation, with RCDs providing selective pollutant recognition coupled with programmable molecular responses and PMDs enabling direct catalytic degradation.

### 2.3. Enzyme-Free Self-Assembly Amplification

Beyond molecular recognition and catalysis, the programmability of DNA enables enzyme-free isothermal amplification (EFIA), a programmable reaction strategy in which toehold-mediated strand displacement drives autonomous hybridization cascades. These EFIA strategies were originally developed to enhance analytical sensitivity by converting a single molecular trigger into amplified DNA outputs [26,46,47]. Beyond signal amplification, certain EFIA strategies can also autonomously assemble higher-order DNA architectures, including extended DNA networks, hydrogels, and functional nanostructures, under mild aqueous conditions [27,48]. The application of this material assembly capability to environmental remediation remains a largely unexplored area, leaving room for the development of new EFIA-based remediation strategies. At the same time, the limited experimental validation in this context highlights the need to establish the functionality and robustness of these systems in highly contaminated and chemically complex environmental matrices before their practical feasibility can be assessed. Nevertheless, this autonomous and programmable assembly capability offers opportunities to extend EFIA from molecular amplification to the construction and dynamic regulation of functional materials for environmental remediation. Among various EFIA strategies, CHA and HCR are the two most widely used systems due to their simplicity, high programmability, and operation under mild isothermal conditions. Both systems induce programmable DNA hybridization chain reactions using toehold-mediated strand displacement without enzymes. While CHA primarily amplifies molecular reactions through the catalytic recycling of hairpin substrates, HCR binds to the initiation strand to sequentially assemble alternating hairpin monomers to form a long double-stranded DNA polymer with a nick [26,49]. Thus, CHA primarily amplifies molecular functions by catalytically generating multiple functional DNA modules, whereas HCR amplifies structural functions by assembling extended DNA polymers and higher-order architectures. The resulting DNA architectures can provide a structural framework for concentrating molecular recognition elements, catalytic DNAzymes, and other functional modules within confined microenvironments. In particular, clamped HCRs (C-HCRs) are useful for constructing functional DNA structures because they introduce multiple arms or clamped DNA components, causing individual HCR products to connect to each other during the polymerization process to form a three-dimensional DNA hydrogel rather than a linear structure (Figure 2D) [50].

Specifically, C-HCR follows the same strand displacement cascade as conventional HCR, consisting of two metastable DNA hairpins (H1 and H2) and an initiator strand (I), but with additional clamping domains engineered into the hairpin monomers. In the absence of the initiator, the complementary domains between H1 and H2 remain sequestered within the folded hairpin structures, preventing non-specific hybridization. Upon introduction of the initiator, toehold-mediated strand displacement opens H1, exposing a previously hidden sequence that subsequently hybridizes with H2. Alternating H1–H2 hybridization then proceeds autonomously, as in conventional HCR. The newly exposed clamping domains simultaneously hybridize with complementary clamping domains on neighboring HCR products, introducing interchain crosslinks that transform one-dimensional DNA polymers into interconnected three-dimensional DNA networks. Importantly, the products of HCR are not merely amplified DNA strands but extended DNA networks that can serve as the structural foundation for functional DNA materials. Unlike isolated DNA strands, higher-order DNA networks enable the collective organization of functional DNA motifs into spatially defined structures. Owing to their porous three-dimensional architecture, high density of programmable functional sites, and compatibility with diverse DNA motifs, these networks provide a versatile framework for integrating molecular recognition, catalytic modules, and other functional components [48,51]. In addition, HCR-mediated assembly can be combined with existing nanomaterials, enabling the assembly of programmable DNA structures onto material surfaces to generate functional DNA nanocomposites with enhanced functional density [52]. Although such amplification of molecular responses and DNA assembly can be advantageous for generating higher-order structures and increasing the density of functional components, it does not necessarily translate into enhanced pollutant removal performance. Further experimental studies are therefore needed to determine whether these amplified responses and resulting structures can effectively improve contaminant capture, sequestration, or degradation. Nevertheless, the extensive use of EFIA in biosensing and environmental monitoring, together with its demonstrated ability to assemble programmable DNA materials, suggests that these systems may provide a useful foundation for developing new environmental remediation strategies. Its ability to translate molecular recognition into amplified molecular responses and higher-order DNA assembly offers the potential to couple contaminant recognition with in situ material formation. Such capabilities may provide opportunities for developing adaptive remediation materials involving target-triggered structural reorganization.

## 3. DNA-Based Material Architectures for Environmental Remediation

While the molecular mechanisms described earlier provide the functional foundation for DNA-based environmental remediation, their practical remediation requires integration into material platforms that provide structural stability and additional useful properties. Owing to its inherent programmability, predictable base pairing, ease of introducing functional groups, and structural diversity, DNA can be rationally designed into various functional architectures such as DNA hydrogels, MNPs, and MSNPs. These material platforms enable DNA-based molecular functions to be incorporated into structurally organized systems while providing additional advantages, such as enhanced structural stability, increased functional density and diversity, high surface area and porosity, magnetic responsiveness, and membrane-based separation capability. This section focuses on the structural characteristics, fabrication strategies, and environmental remediation applications of representative DNA-based material platforms, including hydrogels, porous and magnetic nanomaterials, membranes, and catalytic systems.

### 3.1. Hydrogel Platforms

**Figure 3:**
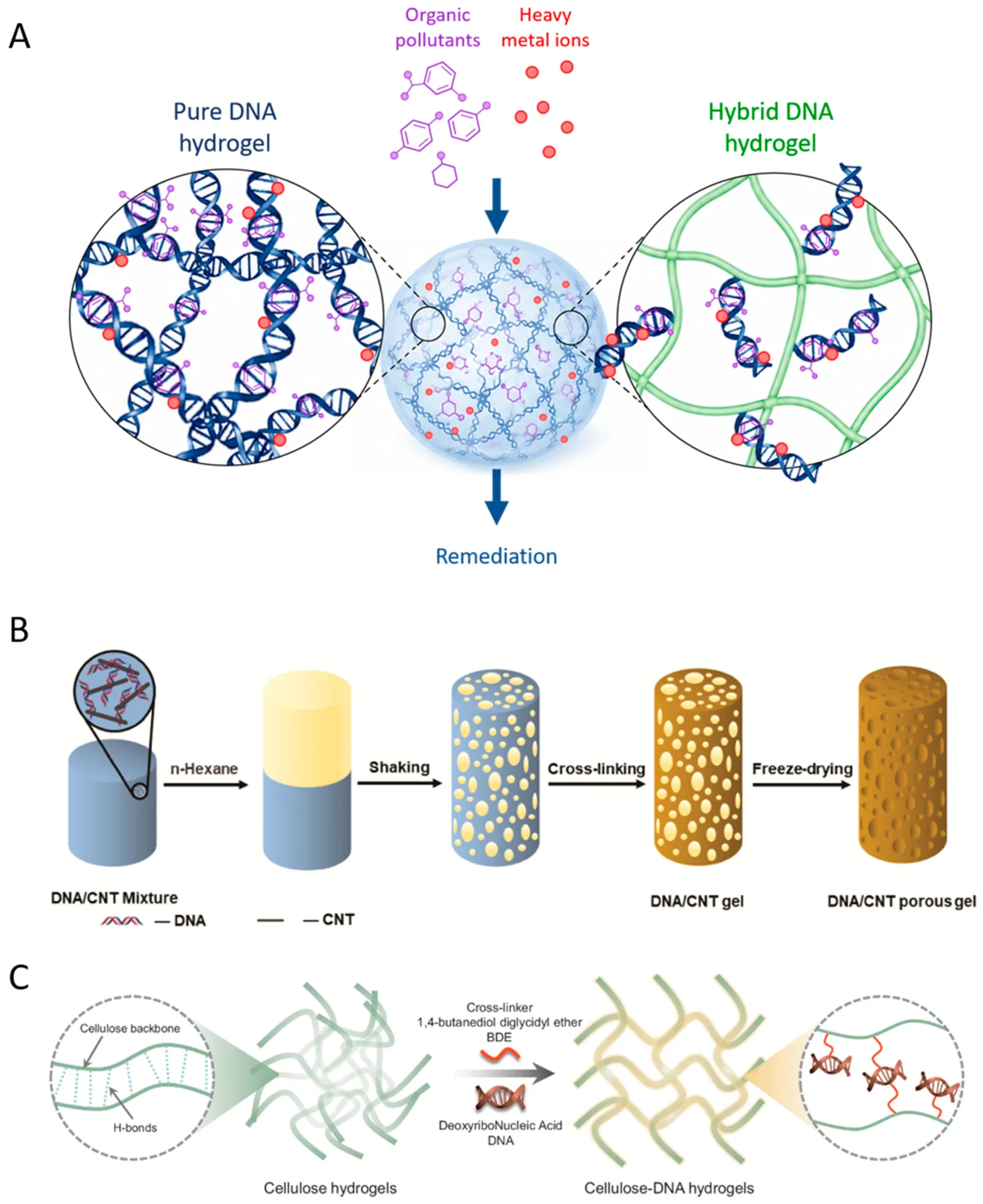
DNA hydrogel-based platforms for environmental remediation. (**A**) Schematic illustration of DNA hydrogel-based remediation through the adsorption of organic pollutants and heavy metal ions, showing pure DNA hydrogels (**left**) and hybrid DNA hydrogels incorporating additional matrix materials (**right**). (**B**) Highly porous DNA/CNT composite hydrogel fabricated using a carbon nanotube-assisted strategy to regulate pore architecture, thereby enhancing the adsorption efficiency for carcinogenic polycyclic aromatic hydrocarbons (PAHs). (**C**) Cellulose/DNA hybrid hydrogel enabling the hydrogen bond-assisted immobilization of enzymes (e.g., laccase) for the efficient capture and catalytic degradation of organic pollutants (panels (**B**,**C**) are adapted from [58,59], respectively). Some graphical elements in panel (**A**) were generated using OpenAI ChatGPT Images 2.0 and subsequently edited and integrated into the final figure by the authors.

DNA hydrogels are three-dimensional cross-linked polymer networks composed wholly or partially of DNA strands that can be applied to hold or capture target substances. These materials offer various advantages, including a porous microstructure, a large surface area, precise nanostructure programming, easy functionalization, programmable stimulus responsiveness, and high stability [13,27,48,51]. Since the first pure DNA hydrogel composed solely of branched DNA building blocks was reported in 2006, DNA hydrogels have been widely studied as biomaterials in the fields of drug delivery, tissue engineering, biosensing, and regenerative medicine [13,27,48,51,53]. More recently, their structural characteristics have attracted increasing interest for their use in environmental applications, particularly as selective adsorbent materials for water remediation [13]. The large surface area and porous network structure of DNA hydrogels facilitate contaminant diffusion, while the densely, negatively charged phosphate backbone provides abundant binding sites for metal ions through electrostatic interactions and coordination, and the aromatic nucleobases contribute to the adsorption of organic contaminants via π–π stacking, hydrogen bonding, and hydrophobic interactions. Current environmental studies generally utilize two DNA hydrogel design strategies: pure DNA hydrogels and hybrid DNA–polymer hydrogels (Figure 3A). The first strategy involves using DNA itself as both a structural support and a functional adsorbent. In 2015, Fernández-Solis et al. demonstrated that DNA-based hydrogels can selectively capture aromatic organic pollutants in aqueous solutions through cooperative π-π stacking interactions, hydrogen bonding, and porous DNA networks. This study demonstrated that DNA hydrogels can function not only as structural biomaterials but also as selective environmental adsorbents capable of removing organic pollutants [54].

The second strategy involves integrating DNA networks into a polymer matrix or reinforcing nanomaterial to overcome the practical limitations of pure DNA hydrogels. While pure DNA hydrogels offer considerable design flexibility through structural programmability and functional versatility, their practical environmental applications are constrained by relatively high material costs, limited mechanical strength, and difficulties in large-scale application [13,51]. To address these limitations, DNA has been incorporated into conventional hydrogel materials or reinforcing nanomaterials, such as chitosan, polyacrylamide, cellulose, and carbon nanotubes (CNTs), to enhance structural stability, recyclability, and economic feasibility while maintaining the DNA’s selective adsorption capabilities. This concept was recognized early by Umeno et al., who incorporated double-stranded DNA into polyacrylamide hydrogels to selectively capture mutagenic aromatic compounds through affinity-based adsorption [55]. Although originally developed for analytical separation rather than environmental remediation, this work demonstrated the feasibility of integrating DNA into polymeric hydrogels while preserving its molecular recognition capability. In a later example, the molecular recognition capability of DNA was combined with the contaminant removal properties of a polymeric hydrogel to integrate detection and remediation functions within a single platform. Dave et al. developed a polyacrylamide hydrogel containing covalently immobilized thymine-rich DNA for the simultaneous detection and removal of Hg^2+^, a representative toxic heavy metal, and demonstrated the system using Hg^2+^-spiked Lake Ontario water [56]. In this system, the immobilized DNA primarily served as a selective recognition element for Hg^2+^ detection through T–Hg^2+^–T coordination, whereas Hg^2+^ removal was predominantly mediated by adsorption to the polyacrylamide matrix. An additional advantage of this hydrogel is that it can be removed after Hg^2+^ adsorption, regenerated by acid treatment, and reused multiple times. Moving toward the more direct use of DNA as a remediation component, Chan et al. developed a DNA–chitosan hydrogel in 2021 in which DNA contributed directly to the adsorptive removal of heavy metal ions, organic dyes, and pharmaceutical contaminants from water [57]. By combining the abundant adsorption sites provided by DNA with the excellent mechanical strength, low cost, and processability of chitosan, the hybrid hydrogel exhibited enhanced structural stability and practical applicability compared with pristine DNA hydrogels. In the same year, Ma et al. reported a highly porous DNA hydrogel utilizing a CNT-based Pickering emulsion, which is an emulsion stabilized by solid particles rather than conventional molecular surfactants (Figure 3B) [58]. This porous structure significantly enhanced the ability to adsorb polycyclic aromatic hydrocarbons (PAHs), which are carcinogenic organic substances. Interestingly, in this study, CNTs not only improved the mechanical properties of the DNA hydrogel but also enhanced adsorption performance by making the DNA hydrogel more porous, thereby increasing the number of binding sites and improving the accessibility of contaminants. This study is an example demonstrating that, beyond molecular programmability, engineering the three-dimensional porous architecture of DNA hydrogels can provide an additional strategy for improving contaminant adsorption. However, the role of previous DNA hydrogels was limited to primarily capturing rather than actively degrading contaminants, and strategies for degrading captured contaminants were not sufficiently presented. More recently, Zhang et al. developed a sustainable cellulose–DNA hydrogel by grafting DNA onto a cellulose matrix, thereby combining the molecular adsorption capability and high loading capacity of DNA networks with the excellent mechanical robustness and sustainability of cellulose [59] (Figure 3C). Furthermore, laccase was immobilized within the hydrogel through charge-assisted hydrogen bonding (CAHB), enabling simultaneous contaminant capture and enzymatic degradation. The resulting bioactive hydrogel efficiently removed a broad spectrum of organic micropollutants while exhibiting excellent recyclability and operational stability, demonstrating the potential of DNA–polymer hybrid hydrogels as multifunctional platforms for practical wastewater treatment.

In summary, the evolution of DNA hydrogel systems reflects a shift from using DNA merely as an adsorptive material to exploiting it as part of multifunctional remediation platforms. Pristine DNA hydrogels demonstrated the feasibility of selective contaminant capture; hybrid hydrogels improved structural robustness, practical applicability, and adsorption capabilities through polymer or reinforcing nanomaterial integration; and bioactive hydrogels further introduced catalytic degradation to couple pollutant enrichment with contaminant elimination. These advancements provide promising design strategies for developing next-generation DNA hydrogel platforms suitable for practical environmental remediation. While these studies demonstrate the feasibility of DNA hydrogels under the aqueous conditions examined, their applicability under more challenging environmental conditions, including extreme pH, elevated ionic strength, and high concentrations of organic contaminants, requires further evaluation. Establishing their operational range under realistic environmental conditions will therefore be important for assessing their suitability for practical remediation. Beyond environmental robustness, another limitation of current DNA hydrogel-based remediation systems is the predominant use of DNA as a passive adsorbent rather than as a programmable functional material. The unique capabilities of functional DNA, including molecular recognition by aptamers, catalytic reactions mediated by DNAzymes, and dynamic molecular or structural responses through enzyme-free DNA circuits, have rarely been incorporated into hydrogel systems for environmental remediation. In other words, while hybrid DNA hydrogels enhance the practicality of DNA-based materials, DNA primarily functions as a structural scaffold or adsorption component in these systems, and its broader programmable capabilities remain largely underexploited. An alternative strategy is to integrate DNA with established environmental materials, allowing DNA to serve as a programmable functional moiety while preserving the intrinsic physicochemical advantages of the supporting materials.

### 3.2. Surface-Functionalized Platforms

Another environmental remediation strategy utilizing DNA is to immobilize functional DNA onto a solid platform. Instead of forming the material itself, the DNA acts as a programmable functional layer, while the underlying substrate retains its original structural and physicochemical properties. As previously mentioned, DNA-based systems have inherent problems such as low mechanical strength, high manufacturing costs, and difficulties in large-scale production. Beyond addressing these issues, this is a highly effective strategy that imparts molecular recognition, selective capture of contaminants, and stimulus response modulation capabilities to DNA while maintaining the inherent properties of the supporting substrate, such as adsorption, filtration, magnetic separation, or structural stability of the solid material [13,14,60,61,62]. One of the earliest examples of integrating DNA with solid supports was reported by Yamada et al., who presented a method to selectively accumulate and remove heavy metal ions by immobilizing DNA onto porous glass beads via UV treatment [63]. This study demonstrated the potential to remove heavy metals in fluidized systems by integrating DNA into solid substrates, such as glass bead columns. This concept evolved to shift the role of DNA introduced into solid support layers from a passive adsorbent to an active molecular recognition element. Instead of utilizing the non-specific affinity of DNA, Hu et al. immobilized aptamers on a chromatographic matrix to introduce target-specific recognition capabilities into existing solid-phase extraction systems [64]. Through this strategy, it was possible to selectively capture and remove trace amounts of pharmaceuticals, such as cocaine and diclofenac, while maintaining the robustness and reusability of the support.

**Figure 4:**
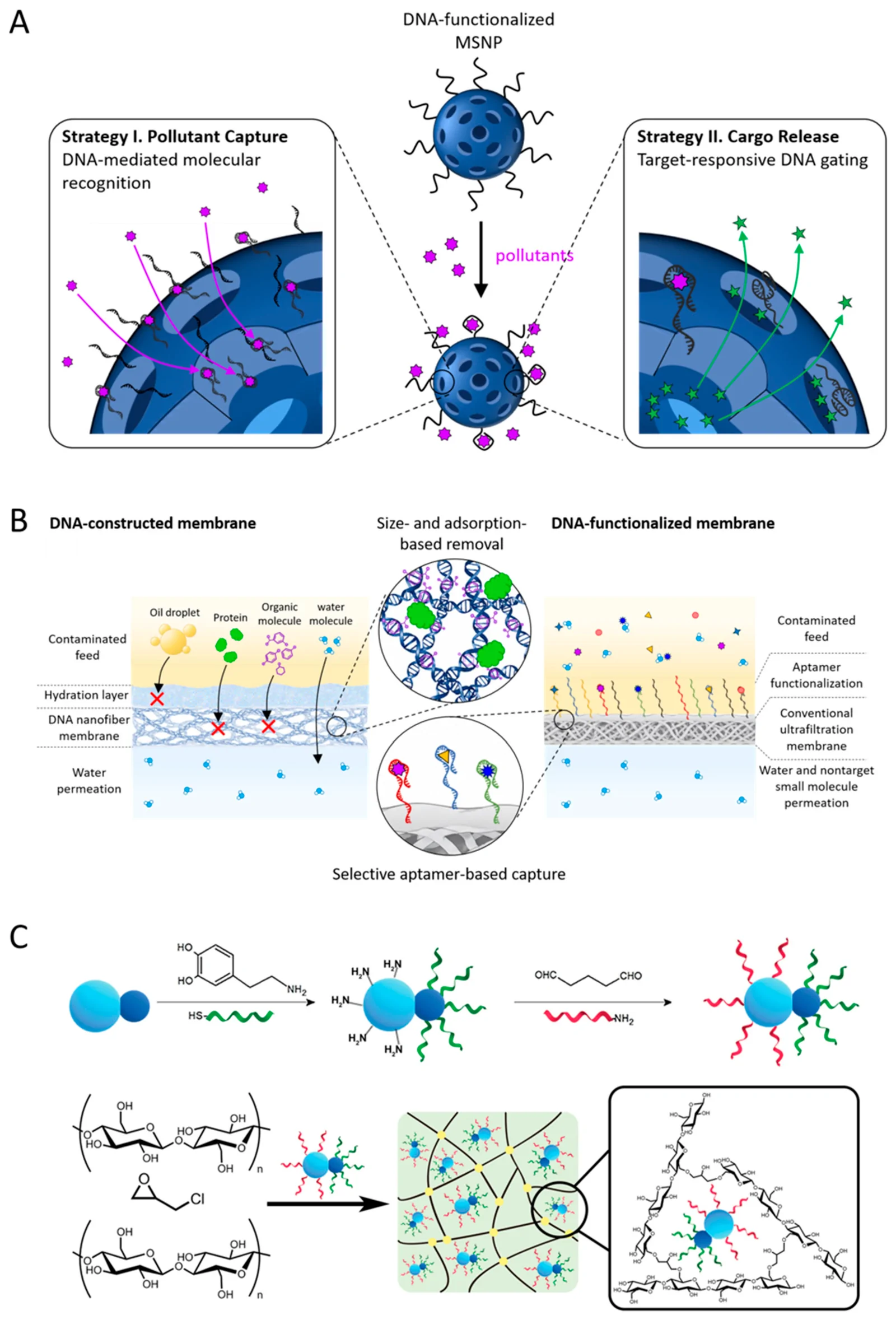
DNA hybrid material platforms integrating the molecular recognition capability of DNA with functional nanomaterials for environmental remediation. (**A**) Schematic illustration of representative DNA-based environmental remediation strategies using MSNPs, including DNA-mediated capture of target pollutants within mesopores (**left**) and target-responsive DNA gating for controlled release of functional cargo (**right**). (**B**) Schematic illustration of DNA-based membrane strategies for environmental remediation. DNA-constructed membranes (**left**) enable contaminant removal through hydration layer-mediated oil repellence, size exclusion, and adsorption, whereas DNA-functionalized membranes (**right**) employ surface-immobilized aptamers for selective recognition and capture of target contaminants. The arrows indicate the direction of pollutant transport, while the cross marks indicate pollutants retained by the membrane. (**C**) A multifunctional Janus nanoparticle platform combining bisphenol A (BPA)-bound aptamer-functionalized MNPs and Hg^2+^-affinity thymine-rich DNA-functionalized silver-based antimicrobial nanoparticles for the simultaneous capture and removal of multiple pollutants (panel (**C**) is adapted from [70]). Some graphical elements in panel (**B**) were generated using OpenAI ChatGPT Images 2.0 and subsequently edited and integrated into the final figure by the authors.

The same design strategy was subsequently extended to other environmental platforms, including porous silica materials, filtration membranes, and MNPs. One of the most widely studied approaches utilizing DNA-integrated complexes in the field of environmental application is the immobilization of aptamers on porous environmental materials, including MOFs and MSNPs. These materials have been extensively studied as versatile adsorbents due to their high surface area, tunable pore structure, and ease of surface functionalization [37,65,66,67]. Although both materials have received significant attention as DNA immobilization platforms in the environmental field, DNA-functionalized MOFs have primarily been studied in environmental monitoring and biosensing, with applications for pollutant remediation remaining a relatively unexplored area; in contrast, DNA-functionalized MSNPs have been successfully applied to the selective removal of environmental pollutants by combining the adsorption properties of mesoporous silica with the programmable molecular recognition properties of DNA (Figure 4A). The mesopores can serve as adsorption spaces where DNA recognition elements selectively capture target pollutants, or as reservoirs for functional cargo whose release is triggered by target-responsive DNA gating. As a representative example of the first strategy, He et al. reported a regenerable, multifunctional core–shell magnetic mesoporous silica nanocomposite for mercury removal [68]. In this system, T-rich DNA capable of selectively recognizing Hg^2+^ was immobilized on MSNPs, enabling the simultaneous detection and selective removal of mercury ions, while the magnetic core facilitated facile recovery of the nanocomposite for subsequent regeneration and reuse. As a representative example of the second strategy, Sudagidan et al. presented a surface sterilization strategy to target and inhibit *Listeria monocytogenes* using aptamer-functionalized MSNPs [69]. Aptamers enhance antimicrobial efficacy and reduce unnecessary disinfectant consumption and secondary environmental pollution by selectively recognizing bacterial cells and guiding nanoparticles to target sites, instead of directly adsorbing contaminants. These studies demonstrate that programmable DNA functions can be integrated into porous adsorbents, including MSNPs.

Membrane-based technologies are widely used in the water treatment field due to their low cost, low energy consumption, high throughput, ease of operation, and scalability [71,72]. However, because conventional membrane separation technologies rely primarily on size exclusion and physicochemical interactions, their molecular selectivity for structurally similar contaminants, particularly those at trace concentrations, is limited [73,74]. Functional DNA can provide strategies to overcome these problems by directly constructing membranes with unique structural and interfacial properties or by introducing programmable molecular recognition capabilities into existing membranes (Figure 4B). As a representative example of the first strategy, Lin et al. reported a cross-linked DNA-based membrane capable of effectively separating oil–water emulsions [75]. This DNA nanofiber network simultaneously served as a membrane framework and a functional separation layer. Owing to its nanoscale fibrous pores and underwater superoleophobicity arising from the hydrated phosphate backbone of DNA, the membrane enabled the simultaneous separation of oil–water emulsions and dissolved organic molecules, proteins, and nanoparticles using a single filtration process. This study demonstrated that DNA itself can function not only as a surface modifier but also as a membrane-forming material for complex wastewater remediation. As a representative example of the second strategy, Romero-Reyes and Heemstra developed an aptamer-based ultrafiltration system capable of selectively removing various low-molecular-weight contaminants from aqueous solutions [76]. Whereas previous aptamer-functionalized membranes were generally designed for a single target, this study incorporated multiple target-specific aptamers into a single membrane to introduce molecular recognition of different contaminants. The resulting membrane enabled the simultaneous removal of atrazine, BPA, and microcystin-LR, even from a complex lake water matrix. This study demonstrated that molecular selectivity for multiple contaminants can be imparted to filtration membranes through the introduction of functional DNA.

For practical environmental remediation, it is necessary to go beyond selective capture and adsorption to include the removal and decomposition of pollutants and the reuse of remediation materials. Magnetic nanomaterials are receiving significant attention in the field of environmental remediation because they enable the rapid removal of various pollutants, such as microplastics, pharmaceuticals, heavy metals, and organic pollutants, through magnetic separation [77,78,79]. A representative example is the renewable Fe_3_O_4_@mesoporous silica nanocomposite for Hg^2+^ removal developed by He et al., as mentioned earlier. In this system, the mesoporous silica provides a large surface area for DNA immobilization, while the magnetic Fe_3_O_4_ core enables the rapid recovery and regeneration of the adsorbent after contaminant removal [68]. Furthermore, more sophisticated particle-based systems are being developed that integrate designed MNPs with various structural and functional components. Wang et al. reported a Janus nanoparticle platform, comprising nanoparticles with two distinct surface regions that can provide different functionalities, in which various aptamers are simultaneously immobilized to recognize and remove diverse contaminants (Figure 4C) [70]. This platform utilizes aptamers, MNPs, antimicrobial particles, and hydrogel structures simultaneously to selectively capture chemically distinct contaminants within a single remediation process, providing magnetic separation and antimicrobial functions. To this end, a Janus nanoparticle system was introduced in which bisphenol A (BPA)-targeted aptamers were functionalized onto MNPs and Hg^2+^-affinity T-rich DNAs were functionalized onto Ag-based antimicrobial nanoparticles. Similar design strategies have also been adopted in hierarchical hybrid nanostructures. For example, Kim et al. reported a remediation platform that integrates molecular recognition, high surface area adsorption, and magnetic separation functions into a single structure in which MNPs are embedded in aptamer-functionalized and silver-coated polydopamine–copper hybrid nanoflowers [80]. In this system, the aptamer provides selective Hg^2+^ recognition, the hybrid nanoflower structure enhances adsorption performance, and the magnetic core enables the rapid recovery and reuse of the adsorbent. Collectively, these studies demonstrate that MNPs serve not merely as recoverable supports but as versatile integration platforms for DNA-functionalized remediation materials. Beyond facilitating magnetic separation and reuse, magnetic cores can serve as versatile platforms for integrating programmable DNA recognition capabilities with other functional materials. Such integration combines DNA-mediated molecular recognition with the physicochemical advantages of magnetic and porous materials, enabling multifunctional remediation platforms that incorporate contaminant capture, sensing, or antimicrobial functions.

Most aptamer-based environmental remediation platforms reported to date are solid supports such as hydrogels, MSNPs, MNPs, and polymer membranes. These supports provide structural robustness, a high surface area, easy recovery, and long-term operational stability. However, immobilization on rigid surfaces limits the morphological flexibility of aptamers, and reduced binding efficiency can result from decreased target accessibility due to steric hindrance and limited molecular diffusion [81]. Furthermore, since the binding performance of aptamers can be influenced by environmental factors such as pH and ionic composition, aptamers immobilized on platforms directly exposed to the external environment may exhibit reduced recognition performance under complex environmental conditions [25,28,82]. To address these requirements, Aptamers-in-Liposomes, a novel compartmentalization platform in which aptamers are encapsulated within the aqueous internal space of liposomes instead of being immobilized on a solid surface, was proposed by Kim et al. in 2011 [82]. This protected microenvironment allows for the selective capture of multiple small organic compounds by encapsulating various aptamer sequences together while maintaining the activity and structural flexibility of the aptamers. Although currently limited to proof-of-concept studies due to difficulties in application to real-world environments compared to solid support-based systems, such as insufficient robustness and recovery challenges, this compartmentalization strategy offers an interesting and promising direction for expanding DNA-based environmental remediation platforms.

### 3.3. Advanced DNA-Based Catalytic Remediation Platforms

**Figure 5:**
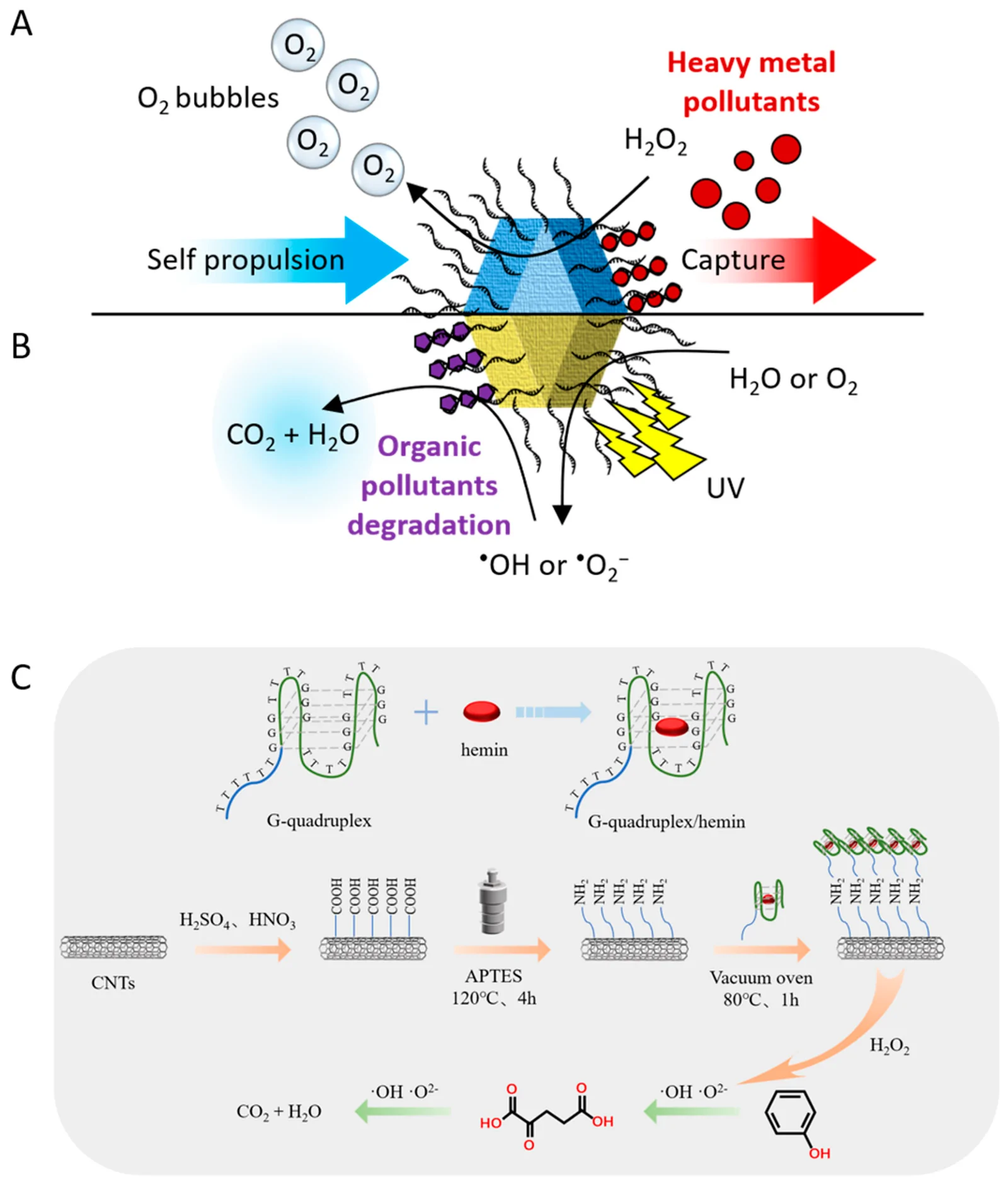
Advanced DNA-based catalytic platforms for environmental remediation. DNA-functionalized catalytic platforms improve contaminant removal (**A**) by enhancing mass transport through catalytic self-propulsion or (**B**) by promoting photocatalytic degradation via reactive oxygen species generation The arrows indicate catalyst-mediated transformations and radical-mediated degradation of organic pollutants, while red circles and purple pentagons represent heavy metal ions and organic pollutants, respectively. (**C**) G-quadruplex/hemin DNAzyme immobilized on amino-functionalized carbon nanotubes to create a recyclable catalytic platform for the degradation of phenolic contaminants (panel (**C**) is adapted from [87]).

Although most DNA-functionalized remediation platforms developed to date rely primarily on selective contaminant capture, recent studies have reported cases combining DNA-mediated molecular recognition with active remediation processes using catalysts [83,84,85,86,87]. DNA-based complexes are being utilized beyond their role as simple passive adsorbents to actively move into contaminated environments or induce catalytic reactions that decompose contaminants. Since mass transfer is often slow under environmentally relevant conditions, autonomously propelled micro/nanomotors have recently emerged as an attractive environmental remediation strategy to overcome these limitations [88]. These motors can actively move through aqueous environments, enhance mass diffusion, and significantly increase the frequency of contact between the remediation material and the target contaminant. Wang et al. developed a catalytically functionalized self-propelled microtube by integrating T-rich DNA, which has an affinity for Hg^2+^, with a catalytic microengine capable of autonomous movement [83] (Figure 5A). This microtube features a cylindrical structure with a Au/Pt bilayer, an internal platinum catalyst decomposes the fuel H_2_O_2_ to generate propulsion, and an external gold layer is introduced to facilitate the functionalization of DNA. While contaminants passively diffuse onto the adsorbent surface in conventional environmental remediation strategies, this self-propelled micromachine moves actively within an aqueous environment to facilitate material transport and increase the frequency of interactions with target substances. This active propulsion not only significantly improves contaminant capture efficiency but also demonstrates the feasibility of combining programmable DNA recognition with autonomous remediation systems.

One of the critical challenges of DNA-based remediation systems is that most currently reported DNA-based remediation platforms are skewed toward contaminant capture. Systems capable of selectively recognizing contaminants and catalytically degrading them on site are attracting increasing attention because they can prevent secondary contamination. DNA-based pollutant degradation strategies have been developed by functionalizing DNA into existing catalytic systems to enhance pollutant degradation capabilities. TiO_2_ is a representative photocatalyst capable of degrading organic pollutants, but its degradation efficiency is limited when there is insufficient contact with the pollutants. Initial studies primarily exploited the intrinsic affinity of DNA to enhance the adsorption of pollutants onto catalyst surfaces, thereby improving photocatalytic degradation efficiency (Figure 5B) [84]. This concept has been further developed, and recent studies have reported using sequence-specific DNA aptamers to selectively concentrate target pollutants at the catalyst interface, thereby imparting target selectivity and concentration effects to the catalyst. Ku et al. functionalized TiO_2_ photocatalysts with target-specific aptamers to overcome the inherent lack of selectivity of conventional photocatalysis [85]. The aptamers selectively enriched target pollutants at the catalyst interface, resulting in enhanced photocatalytic degradation efficiency while simultaneously reducing the toxicity of the degradation products. This work demonstrates the potential of integrating molecular recognition with catalytic remediation for selective pollutant degradation. This strategy combining target specificity and catalytic degradation can also be applied to other advanced oxidation processes (AOPs), such as nano-Fenton systems, to further enhance degradation selectivity and efficiency. Liu et al. reported a nano-Fenton system that effectively catalytically degrades targets using target-specific aptamers [86]. In this study, we enabled an immediate Fenton oxidation reaction through a recognition–degradation mechanism by spatially localizing target pollutants to the catalytic active site, Fe-Au, using aptamers. By combining molecular recognition-mediated target enrichment with highly reactive catalytic degradation, the aptamers functioned not only as capture ligands but also as regulators determining the location of oxidation reactions, dramatically improving degradation selectivity in complex environmental matrices. In addition to chemical catalysts, DNA has also been integrated with protein enzymes to enhance catalytic remediation. For example, the cellulose/DNA hydrogel described above was further functionalized with laccase, combining broad-spectrum pollutant adsorption with enzymatic degradation to improve remediation efficiency [59].

Beyond DNA-assisted catalytic systems, DNA itself can be directly utilized as a catalyst using the aforementioned DNAzyme. Unlike aptamer-assisted catalytic platforms, where DNA primarily serves as a molecular recognition element to localize target contaminants at catalytic active sites, DNAzyme actively participates in catalytic reactions, offering the potential to directly combine programmability with contaminant transformation. Ren et al. reported a representative proof-of-concept case in which peroxidase-like G-quadruplex/hemin DNAzyme was immobilized on amino-functionalized carbon nanotubes (CNT–NH_2_) to construct a recyclable catalytic platform for phenol degradation (Figure 5C) [87]. By immobilizing DNAzyme on CNTs, catalytic activity, stability, and reusability were significantly improved compared to those of free DNAzyme, enabling the efficient degradation of phenol through H_2_O_2_-mediated oxidation. Although this study is a proof of concept and its application is currently limited to model organic contaminants, it is one of the few examples demonstrating the direct catalytic use of DNAzymes for oxidative pollutant degradation, in contrast to their more established use as functional components in biosensing systems. Despite this demonstration, DNAzyme-based catalytic remediation remains largely at the proof-of-concept stage, with only a limited number of studies directly exploiting DNAzyme catalysis for pollutant degradation. To date, DNAzymes have primarily been used for pollutant detection, and instances of integrating catalytic potential for pollutant degradation or active remediation into DNAzyme-based remediation systems are still rare. Furthermore, although nanozymes have been extensively investigated for pollutant degradation, DNA-functionalized nanozyme systems have been developed primarily for biosensing applications, while their direct application to environmental remediation remains limited [89,90,91]. Integrating these individual studies into an environmental remediation platform can present a promising path toward highly selective, catalytic, and programmable pollutant remediation systems that go beyond existing adsorption-based strategies.

## 4. DNA-Based Remediation Strategies for Different Classes of Environmental Pollutants

The various DNA-based environmental remediation platforms discussed in the previous section have broadened the range of technologies applicable to the field of environmental remediation and have significantly extended the range of pollutants that can be selectively removed, ranging from heavy metal ions to organic pollutants, pharmaceuticals, and pathogens. DNA-based systems utilize various molecular mechanisms, such as intrinsic adsorption based on nucleic acid structure, sequence-specific molecular recognition by aptamers, and catalytic degradation by DNAzymes or DNA-induced catalytic systems. While many DNA-based remediation platforms target various types of contaminants, this section summarizes DNA-based remediation systems for heavy metal ions, organic dyes, pharmaceuticals, pesticides, emerging contaminants, and biological contaminants, classified by type of contaminant, and examines the environmental remediation strategies utilizing DNA materials.

### 4.1. Heavy Metal Ions

Heavy metal ions are the most extensively studied subjects in the field of environmental remediation due to their high toxicity, persistence, and bioaccumulation [92]. Because heavy metal contaminants are difficult to decompose, their removal relies primarily on physical separation through adsorption, precipitation, ion exchange, or membrane filtration [93]. To date, DNA-based remediation strategies for various heavy metal ions using various DNA functions and material platforms have been reported. Table 1 summarizes representative studies on heavy metal ions based on target contaminants, DNA functions, and binding mechanisms.

Among various environmental pollutants, heavy metal ions have been the most extensively investigated targets for DNA-based remediation because the programmable molecular interactions of DNA enable two distinct remediation strategies: broad-spectrum adsorption through the intrinsic physicochemical properties of DNA and sequence-specific metal capture through engineered nucleic acid sequences. Early DNA-based heavy metal removal research utilized DNA itself as an adsorbent for various heavy metal ions rather than as molecular recognition elements. Owing to its abundant negatively charged phosphate backbones and electron-donating bases, DNA can bind to various metal ions through electrostatic interactions and metal–ligand coordination [57,63,94]. One early study applying these DNA capabilities to the field of environmental remediation was the aforementioned study by Yamada et al., who reported that insolubilizing DNA functionalized on porous glass beads effectively accumulates several heavy metal ions, including Hg^2+^, Pb^2+^, Cd^2+^, and Cu^2+^, in an aqueous environment [63]. This study confirmed that DNA itself possesses metal adsorption capabilities without requiring engineered sequences and that these capabilities could be retained after incorporation into a solid support. Subsequent research utilized the unique adsorption properties of DNA by integrating them into various supports. For example, Yang et al. developed DNA-encapsulated polyethersulfone (PES) hollow microspheres as a reusable composite adsorbent capable of simultaneously removing heavy metal ions and aromatic organic compounds [94]. Subsequent DNA-based heavy metal removal studies interestingly focused on programmable molecular recognition elements, including aptamers and DNAzymes, rather than the intrinsic broad-spectrum adsorption capability of DNA. It was only recently that a DNA–chitosan hydrogel was reported by Chan et al., which expanded the range of adsorbable contaminants by combining the inherent adsorption capacity of DNA with the additional function of chitosan [57]. This paper demonstrated that, in addition to the existing coordination of phosphate groups and nucleobases, the chelation of the amine group of chitosan can effectively remove environmentally important Hg^2+^, Pb^2+^, Cd^2+^, and Cu^2+^ while also expanding the scope to various types of pollutants.

Another heavy metal removal strategy, sequence-specific recognition, can achieve much higher selectivity for specific heavy metal ions than the aforementioned intrinsic adsorption. This specificity is very important because environmentally important metal ions often coexist in large quantities with chemically similar competing metal ions. To achieve selective recognition, the engineered DNA sequence utilizes sequence-specific mechanisms such as metal-mediated base pairing or metal ion aptamers to provide much higher affinity and specificity for various heavy metal ions including Hg^2+^, As(III), As(V), Mn^2+^, Pb^2+^, Co^2+^, and UO_2_^2+^ [56,68,70,80,83,95,96,97,98,99,100,101,102,103]. Among heavy metal contaminants, mercury is unique in its ability to form highly stable metal-mediated base pairs with thymine mismatches [104]. This is attributed to the ability of Hg^2+^ to coordinate with two opposing thymine bases, thereby acting as a coordination bridge that stabilizes the T–Hg^2+^–T metal-mediated base pair. T-rich DNA for Hg^2+^ capture offers several advantages, such as simple sequence design without the need for SELEX, highly selective Hg^2+^ binding, and the presence of multiple thymine residues providing multiple potential Hg^2+^ binding sites within a single DNA strand. Furthermore, mercury is receiving particular attention because it exhibits severe neurotoxicity even at trace concentrations and requires highly selective removal due to strict discharge regulations. For practical remediation, however, the selective Hg^2+^ recognition of T-rich DNA needs to be integrated with suitable material platforms that enable the captured mercury to be separated and recovered from the treated water. Accordingly, thymine-rich DNA has been extensively integrated with robust porous materials, such as polyacrylamide hydrogels, MSNPs, and nanoflower architectures, as well as multifunctional materials, including graphene, MNPs, silver nanoparticles (AgNPs), and self-propelled micromotors, to achieve the selective capture and efficient removal of mercury [56,68,70,80,83,95].

Meanwhile, metal ion aptamers selectively bind to target ions through sequence-dependent folding into well-defined three-dimensional conformations, thereby forming specific binding pockets for target recognition [28]. These systems exhibit a high binding affinity for their target ions while minimizing interference from competing metal species. Although environmental applications of aptamers have predominantly focused on monitoring, several studies have experimentally demonstrated their use for the selective removal or separation of environmentally important heavy metals, such as arsenic and lead, which are highly toxic even in trace amounts, or manganese, which causes disease through excessive accumulation. For example, arsenic-binding aptamers reported by Kim et al. demonstrated potential for selective groundwater remediation, while manganese-specific aptamers reported by Jang et al. showed applicability in reservoir water treatment [96,97]. In addition, Li et al. demonstrated the selective separation and preconcentration of trace lead from environmental water samples using aptamer-functionalized magnetic composites [98]. However, despite the successful development and widespread application of aptamers for numerous heavy metal ions, including Cd^2+^ and Pb^2+^, in environmental monitoring, instances of their direct application to contaminant removal and separation remain relatively limited [28,105]. Although representative heavy metal-binding aptamers can exhibit high binding affinities, with reported Kd values spanning the nanomolar to micromolar range depending on the target and aptamer sequence [105], a high binding affinity does not necessarily translate into a high binding capacity. One possible explanation is that the binding capacity of individual aptamer molecules is inherently limited by the number of available target-binding sites, restricting the number of target ions that can be captured by each aptamer [105]. Consequently, increasing removal capacity requires a correspondingly large amount of functional DNA. In contrast, the same aptamers can serve as highly efficient molecular switches to initiate downstream signal amplification reactions, making them considerably more advantageous for environmental monitoring than for large-scale contaminant removal.

Interestingly, studies utilizing aptamers for the selective separation and recovery of metal ions in aqueous environments have also expanded to applications with distinct objectives beyond conventional toxic metal removal. Lee et al. developed cobalt-binding aptamer-functionalized beads for the selective removal and recovery of radioactive cobalt from liquid radioactive waste [99]. In this case, the application of selective metal ion capture was extended from conventional heavy metal removal to the decontamination of radioactive metal species in liquid radioactive waste, where the captured radionuclides remain radioactive, requiring additional consideration of the stability of DNA-based materials under radiological conditions and the subsequent management of radionuclide-loaded materials. Another extension of DNA-mediated selective capture to radioactive metal species has been demonstrated in studies targeting the selective separation and recovery of UO_2_^2+^ present at very low concentrations in complex aqueous environments such as seawater [100,101,102,103]. Although uranium is a radioactive element, it is also a valuable nuclear resource; therefore, the recovery of uranium from seawater is primarily regarded as selective resource recovery rather than radionuclide decontamination. Together, these studies demonstrate that DNA-mediated selective metal recognition can support distinct applications ranging from environmental remediation and radioactive waste treatment to selective resource recovery.

Collectively, these studies indicate that DNA-based heavy metal remediation has been most actively explored for Hg^2+^ removal and UO_2_^2+^ recovery. Although the intrinsic, non-specific metal-binding capability of DNA enables the adsorption of a broad range of heavy metal ions, relatively few studies have further developed this strategy for direct environmental remediation. This limited development may partly reflect the relatively modest advantages of intrinsic DNA adsorption over conventional adsorbents when molecular selectivity is not required, particularly in terms of material cost and adsorption capacity. In contrast, programmable and sequence-specific molecular recognition represents a major advantage of DNA and has been extensively exploited in environmental monitoring, although its translation into contaminant removal remains constrained by the relatively low binding capacity of many metal-binding aptamers. Nevertheless, exceptional examples, including T-rich DNA for Hg^2+^ capture and uranium-binding aptamers for selective resource recovery, demonstrate that DNA-mediated molecular recognition can become a highly effective remediation strategy when coupled with either enhanced adsorption capacity or additional application value, such as resource recovery.

### 4.2. Organic Contaminants

Organic pollutants encompass a wide range of carbon-based chemicals that are persistent, accumulative, and toxic, including dyes, pharmaceuticals, pesticides, biotoxins, pesticides, and other emerging pollutants [106,107]. Since these pollutants can be chemically converted into low-toxicity intermediates or completely mineralized into CO_2_ and H_2_O, catalytic degradation is an attractive remediation strategy. Conventional treatment technologies, including adsorption, biological treatment, photocatalysis, and AOPs, have therefore been extensively investigated for organic contaminant removal [107,108,109]. Consequently, depending on the target pollutants and remediation objectives, DNA can directly or indirectly promote pollutant degradation by performing various roles; for example, they can act as adsorbents, sequence-programmed recognition elements, or catalytic components. Therefore, the remediation of organic pollutants using DNA involves a wider range of mechanisms than those used for heavy metal removal. Table 2 summarizes representative studies on organic contaminants based on remediation mechanisms, pollutant categories, and DNA functions.

Similar to heavy metal ions, organic pollutants can also be captured by DNA through two distinct molecular interaction mechanisms. One is broad adsorption due to the inherent physicochemical properties of DNA, and the other is highly selective recognition by sequence-specific aptamers. The former is based on the inherent affinity that DNA has for organic molecules, which arises from various non-covalent bonds such as π-π stacking, electrostatic interactions, hydrogen bonding, and van der Waals interactions, and these interactions enable various binding modes such as insertion, groove binding, and external adsorption depending on the physicochemical properties of the target molecule [110]. Planar bases of double-helix DNA can interact with planar aromatic compounds through π-π stacking, while the negatively charged phosphate backbone and hydrogen-bonding interactions further contribute to pollutant adsorption. For example, the anthraquinone dye furfurin has been shown to bind to double-stranded DNA through a combination of π–π stacking, hydrogen bonding, and groove bonding, demonstrating that it can effectively capture aromatic organic molecules using only unmodified DNA [111]. Furthermore, Fernández-Solis et al. compared linear DNA with a method utilizing DNA itself as a hydrogel to evaluate the efficiency of removing aromatic organic pollutants [54]. They demonstrated that pollutant affinity was strongly influenced by the double-helical content of DNA, while the hydrogel format provided a practical advantage by eliminating the additional precipitation or condensation steps required to recover soluble DNA after pollutant binding. In subsequent studies, based on these intrinsic interactions, DNA was incorporated into various functional materials, including hydrogels, membranes, and porous microspheres, to improve DNA stability, facilitate the recovery and reuse of adsorbents, and achieve the broad-spectrum removal of organic pollutants [55,57,58,59,94,112]. For example, in the case of hydrogels, dsDNA-containing polyacrylamide hydrogels efficiently adsorbed mutagenic aromatic compounds, while DNA–chitosan hydrogels simultaneously removed organic dyes, pharmaceuticals, and heavy metal ions; DNA/CNT hydrogels effectively removed carcinogen PAHs through their highly porous structure; and cellulose-DNA hydrogels were used to remove organic dyes and phenolic organic pollutants [55,57,58,59]. In the case of solid supports, DNA-encapsulated PES microspheres and DNA-immobilized magnetic chitosan composites exhibited high adsorption capacities for various organic dyes and aromatic contaminants, demonstrating that DNA itself can function as a versatile affinity material for pollutant capture [94,112]. These broad-spectrum DNA adsorbents offer several advantages, including a simple sequence design and the ability to simultaneously capture a wide range of organic pollutants. However, their limited molecular selectivity may make them susceptible to competitive adsorption by coexisting natural organic matter and structurally similar compounds in complex environmental matrices, potentially limiting their effectiveness in the selective removal of trace toxic contaminants.

Interestingly, the DNA-based remediation strategies reported for organic pollutants differ distinctly depending on the characteristics of the target contaminants. Aromatic contaminants with a high affinity for DNA, such as PAHs and organic dyes, are primarily removed through the intrinsic affinity of DNA, whereas emerging organic contaminants present in trace amounts in complex environmental matrices and requiring high selectivity, such as BPA, atrazine, pharmaceuticals, illicit drugs, and mycotoxins, are primarily removed using target-specific aptamers [64,70,76,82,113,114,115,116,117,118]. While broad adsorption involves the DNA itself acting as the adsorbent, aptamers function as programmable molecular recognition elements that achieve selective target capture. For environmental remediation, the efficient recovery and reuse of the remediation platform are important considerations. Because free aptamers are difficult to separate from treated water after pollutant capture, they are commonly immobilized onto a variety of supporting materials. Such immobilization can facilitate material recovery and reuse while improving structural stability. Accordingly, aptamers have been integrated into a variety of functional supports, including MNPs, membranes, ultrafiltration systems, and porous materials, to achieve selective pollutant capture while facilitating material recovery and continuous water remediation [64,70,76,115,116,117,118]. The choice of support material is determined by the required remediation process. MNPs facilitate rapid recovery using magnetic separation, whereas membrane and filtration-based platforms are more suitable for continuous-flow remediation because immobilized aptamers remain fixed within the treatment module while the treated water passes through. Whereas typical aptamer-based environmental remediation platforms rely on surface immobilization, aptamers have also been encapsulated within liposomes to create optimized recognition environments that better preserve their conformational flexibility and molecular recognition capability [82].

Since organic pollutants, unlike heavy metals, can potentially be transformed into less harmful products through catalytic degradation, this provides opportunities for remediation strategies that extend beyond physical capture. Catalytic degradation strategies have been developed primarily for environmentally important persistent organic pollutants, such as endocrine-disrupting chemicals, pharmaceuticals, pesticides, and mycotoxins, for which catalytic transformation or degradation may offer environmental benefit rather than simple adsorption [119]. In addition, catalytic degradation offers the additional benefit of permanently removing pollutants and preventing their re-release during the adsorbent regeneration or disposal process. While intrinsic adsorption and aptamer recognition differ in molecular selectivity, both strategies can concentrate pollutants near catalytic interfaces, providing a basis for coupling pollutant capture with subsequent degradation. Based on these concepts, DNA can be integrated into existing catalytic systems to link pollutant capture with subsequent degradation. Suzuki et al. demonstrated that immobilizing DNA adsorbents onto TiO_2_ photocatalysts can concentrate DNA-affinitive organic pollutants at the catalytic interface, thereby enhancing their photocatalytic degradation [84]. Similarly, Ku et al. functionalized TiO_2_-based photocatalysts with aflatoxin B_1_ (AFB_1_)-specific aptamers, enabling the selective degradation of low concentrations of AFB_1_ in a complex peanut oil matrix [85]. Importantly, the resulting degradation products exhibited significantly reduced toxicity. Based on this concept, Liu et al. developed an aptamer-assisted nano-Fenton system capable of selectively degrading novel pollutants, including BPA, oxytetracycline (OTC), and dibutyl phthalate (DBP), in complex water environments, while Zhang et al. developed a laccase-immobilized cellulose–DNA hydrogel capable of degrading PFASs, antibiotics, organic dyes, and PAHs [59,86]. These studies demonstrate that pollutant capture utilizing DNA can also serve as an effective strategy to enhance catalytic degradation efficiency. Finally, DNA itself can directly function as a catalyst through DNAzymes. A representative example is the G-quadruplex/hemin DNAzyme, which exhibits peroxidase-like activity for phenol degradation [87]. Overall, DNA-based remediation of organic pollutants has diversified into complementary strategies targeting various types of pollutants. Broad adsorption is suitable for aromatic compounds that inherently exhibit DNA affinity, whereas target-specific aptamers are suitable for emerging organic pollutants present in trace amounts within complex environments. Furthermore, since both broad adsorption and target-specific aptamers can be combined with subsequent catalytic degradation, they can provide a potential strategy for extending DNA-based remediation from pollutant capture to subsequent degradation across a broader range of organic contaminants.

In summary, these studies demonstrate the broad applicability of DNA-based remediation technologies to chemically diverse environmental contaminants, ranging from heavy metal ions to organic pollutants. Whereas DNA-based remediation of heavy metal ions has primarily evolved into efficient capture and recovery through intrinsic adsorption and sequence-specific recognition, remediation of organic pollutants has further expanded beyond pollutant capture to incorporate catalytic degradation, enabling irreversible detoxification of persistent contaminants.

**Table 2:** Summary of representative DNA-based organic pollutant remediation studies.

Remediation Mechanism	Target Organic Pollutant Category	Function	References
Broad-spectrum adsorption (π–π stacking, intercalation, electrostatic interaction)	PAHs	Affinity adsorbent	[54,58]
	Organic dyes		[57,59,94,111,112]
	HAAs		[55],
	Pharmaceuticals		[57]
Selective recognition	Pesticides	Aptamer	[76]
	EDCs (e.g., BPA and 17β-estradiol)		[70,76,82,115,116]
	Biotoxins		[76,113,114,117,118]
	Pharmaceuticals		[64,82]
	Illicit drugs		[64]
Catalytic degradation	PAHs, PFASs, pharmaceuticals	Affinity adsorbent	[59]
	Organic dyes		[84]
	AFB_1_	Aptamer	[85]
	BPA, OTC, DBP		[86]
	Phenols	DNAzyme	[87]

Abbreviations: PAHs, polycyclic aromatic hydrocarbons; HAAs, haloacetic acids; EDCs, endocrine-disrupting chemicals; BPA, bisphenol A; PFASs, per- and polyfluoroalkyl substances; OTC, oxytetracycline; DBP, dibutyl phthalate; AFB_1_, aflatoxin B_1_.

### 4.3. Biological Contaminants

Beyond chemical pollutants, biological contaminants, including pathogenic microorganisms, viruses, endotoxins, and antimicrobial resistance genes (ARGs), have recently emerged as an important category of environmental contaminants because of their persistence in water systems and their potential risks to both ecological safety and public health [120,121]. Unlike heavy metals and organic contaminants, many biological contaminants possess the unique ability to replicate, spread, and evolve, allowing even a small initial contamination event to develop into large-scale outbreaks [121]. Disinfection is the process of eliminating, removing, or inactivating pathogenic microorganisms and typically relies on non-specific inactivation mechanisms, including chlorination, ozonation, ultraviolet (UV) irradiation, and thermal sterilization. While these approaches are effective, they have several limitations, including incomplete removal, the removal of non-target microorganisms, the formation of undesirable byproducts, damage to the surrounding ecosystem, and reduced efficacy against resistant pathogens and proteinaceous infectious agents. One promising strategy to overcome these limitations is affinity-based decontamination, in which target biological contaminants are selectively captured prior to their subsequent removal or inactivation. To meet these requirements, DNA aptamers, which are capable of selectively recognizing viruses, bacteria, and pathogenic proteins, represent a promising molecular recognition platform for biological decontamination. Table 3 summarizes representative studies on biological contaminants based on remediation mechanisms, target biological contaminants, and the role of DNA. A notable feature is that, unlike heavy metals and organic pollutants, selectivity for biological contaminants is almost exclusively achieved through aptamer-mediated molecular recognition, whereas the subsequent elimination step is typically provided by functional supporting materials.

Owing to their high target specificity and binding affinity, aptamers have been extensively developed for biosensing and biomedical applications. In contrast, their direct application to the selective removal and inactivation of biological contaminants in environmental remediation remains relatively limited. Nevertheless, studies in environmental decontamination and related biomedical and antimicrobial fields have experimentally demonstrated diverse strategies for aptamer-mediated capture and selective pathogen elimination, providing a foundation for the further development of DNA-based environmental decontamination. In particular, some biomedical proof-of-concept studies have demonstrated the potential of aptamer-mediated recognition for selective viral capture and removal. For example, affinity adsorbents functionalized with aptamers targeting the hepatitis C virus (HCV) genotype 2a envelope glycoprotein (E1E2) selectively captured and removed HCV particles [122]. Similarly, Zheng et al. immobilized hepatitis B virus surface antigen (HBsAg)-binding aptamers on agarose using a DNA tetrahedron as an intermediate structure, enabling efficient affinity-based removal of HBsAg from human plasma [123]. Collectively, these studies demonstrate the feasibility of translating aptamer-mediated molecular recognition beyond sensing into the selective capture and removal of biological targets, although its application to environmental decontamination remains largely unexplored. Beyond affinity capture, DNA-mediated recognition strategies have been further extended to pathogen elimination by integrating aptamer-mediated recognition with functional antimicrobial materials. For example, Sudagidan et al. developed aptamer-functionalized MSNPs for targeted disinfection, in which selective recognition of *Listeria monocytogenes* enabled localized delivery of the antimicrobial agent benzalkonium chloride (BAC) and effective bacterial inhibition at reduced disinfectant doses [69]. Building upon targeted antimicrobial delivery strategies, more sophisticated DNA nanostructures have recently emerged as programmable antibacterial platforms. For example, Wu et al. developed a bacteria-specific aptamer-functionalized DNA tetrahedron co-loaded with ciprofloxacin (CIP) and AgNPs, enabling the efficient elimination of antibiotic-resistant bacteria through synergistic antibacterial therapy [124]. This work demonstrates that programmable DNA nanostructures can integrate bacterial recognition, structural programmability, and the co-delivery of multiple therapeutic agents within a single nanoscale platform, highlighting their potential for adaptation to the selective elimination of biological contaminants.

Beyond these antimicrobial delivery strategies, DNA-mediated recognition has also been integrated with photocatalytic and photothermal mechanisms for selective bacterial elimination. Although photocatalysts have been widely employed for the oxidative degradation of organic pollutants through the generation of reactive oxygen species (ROS) under light irradiation, they can also be utilized for antibacterial applications by inducing localized oxidative damage to microbial cells. In this regard, Song et al. reported a TiO_2_ photocatalyst functionalized with an *Escherichia coli* (*E. coli*) surface-specific single-stranded aptamer cocktail, which enabled the selective recognition of target bacteria while simultaneously enhancing photocatalytic antibacterial activity through localized ROS generation [125]. Another related strategy is the use of photothermal therapy, in which light is converted into heat to eradicate microorganisms. Ocsoy et al. reported a rapid strategy for the selective elimination of methicillin-resistant *Staphylococcus aureus* (MRSA) using an aptamer-conjugated magnetic graphene oxide platform that specifically recognizes MRSA [126]. In this system, graphene oxide served as a photothermal agent, generating localized heat upon near-infrared (NIR) laser irradiation, while MNPs grown on the graphene oxide surface enabled magnetic enrichment of MRSA under an external magnetic field. Together, these studies demonstrate that aptamer-mediated bacterial recognition can be coupled with photocatalytic or photothermal processes for selective bacterial elimination, although the translation of such strategies into broader environmental remediation applications remains limited.

While the examples above illustrate how DNA recognition can directly contribute to pathogen elimination, programmable DNA also offers a complementary strategy by augmenting existing remediation materials with additional target-specific recognition capabilities. Such integration enables broader contaminant coverage while preserving the intrinsic remediation functions of the original materials. For example, aptamer-functionalized ultrafiltration systems have demonstrated that by integrating programmable DNA recognition into existing membrane separation platforms, selective molecular capture of additional target contaminants can be introduced while maintaining the intrinsic filtration function of PEG membranes for the physical removal of microorganisms and suspended particles [76]. The same design principle has also been applied to hydrogel-based materials. Wang et al. enabled the capture of additional contaminants by introducing aptamers while maintaining the function of existing cellulose hydrogels for capturing *E. coli* [70]. These studies demonstrate that DNA can complement existing remediation materials by introducing programmable target specificity, rather than enhancing or replacing existing remediation mechanisms.

In summary, these studies reported thus far demonstrate that the programmability of DNA can be fundamentally extended to the remediation of biological contaminants through aptamer-mediated recognition and its integration with diverse antimicrobial and separation mechanisms. However, the application of programmable DNA to environmental remediation of biological contaminants remains less explored than its use in biosensing and diagnostic applications. In contrast, DNA aptamers have already been extensively developed to recognize a wide range of biological targets, including pathogenic bacteria and viruses [127,128]. Bridging this gap represents an important opportunity for the further development of DNA-based remediation, as the extensive repertoire of existing aptamers could potentially be integrated into platforms for the selective capture and elimination of diverse biological contaminants. Rather than requiring entirely new molecular recognition elements, future DNA-based biological remediation may therefore benefit from repurposing the extensive aptamer libraries that have already been established for biosensing and diagnostic applications.

## 5. Challenges and Future Perspectives

Although remarkable progress has been achieved in DNA-based environmental remediation by exploiting the exceptional programmability and molecular recognition capabilities of DNA, several fundamental challenges, including limited environmental stability, susceptibility to nuclease degradation, high production costs, and difficulties associated with large-scale manufacturing and practical implementation, continue to hinder the widespread application of these systems. In addition, current DNA-based remediation studies have predominantly focused on aqueous environments, and extending these systems to more complex environmental matrices, including soils, sediments, sludge, and subsurface environments, remains an important challenge for broader environmental implementation. Beyond addressing these practical limitations and expanding the range of applicable environmental matrices, current DNA-based remediation technologies remain at an early stage of development, utilizing only a small fraction of DNA’s functional and structural potential. Indeed, many DNA-based technologies that have been extensively explored and validated in other fields have yet to be translated into environmental remediation applications. Therefore, important directions for future research include expanding the functional diversity of DNA, extending the material scope of DNA-based remediation systems through the rational integration of emerging functional materials, and broadening the range of environmental contaminants that can be effectively addressed. Accordingly, this section outlines future perspectives for translating DNA-based environmental remediation into practical applications by addressing the intrinsic limitations of DNA while expanding its functional diversity, broadening the material scope of DNA-integrated remediation systems, and extending the spectrum of environmental contaminants that can be effectively remediated.

### 5.1. Future Directions of DNA-Based Remediation

Over the past two decades, DNA-based environmental remediation has evolved from simple proof-of-concept studies into a rapidly expanding research field. As discussed in the previous sections, the unique programmability of DNA and its integration with advanced functional materials continue to reveal substantial opportunities for future remediation technologies. Nevertheless, several fundamental challenges must still be addressed before these systems can be translated from laboratory demonstrations into practical environmental remediation technologies. Most reported DNA-based platforms have been evaluated under simplified laboratory conditions using model pollutants and controlled aqueous environments, whereas real environmental systems are considerably more complex, containing fluctuating pH, ionic strength, natural organic matter, microorganisms, competing contaminants, and continuous water flow. These factors may significantly influence DNA stability, target accessibility, molecular recognition efficiency, and overall remediation performance [82,129]. Therefore, a major goal for the future development of DNA-based remediation systems is to retain the high remediation performance demonstrated under ideal laboratory conditions when operating in complex real-world aqueous environments by developing strategies that mitigate the detrimental effects of variable environmental conditions.

In addition to addressing these challenges within aqueous environments, expanding DNA-based remediation to other environmental matrices represents another important challenge for broader environmental implementation. To date, DNA-based remediation studies have predominantly focused on aqueous systems, while their application to more heterogeneous matrices such as soils, sediments, sludge, and subsurface environments remains relatively limited. Compared with aqueous systems, these matrices present additional complexities arising from their heterogeneous physicochemical properties and structures, which can strongly influence contaminant distribution, mobility, and accessibility [130,131]. These effects may limit interactions between target contaminants and DNA-based recognition or adsorption sites, potentially reducing the efficiency and predictability of pollutant capture under such complex environmental conditions. Moreover, interactions of DNA with soil colloids, mineral surfaces, organic matter, and other solid components may alter its accessibility, conformation, mobility, and environmental stability [132]. Such effects may be particularly important for aptamer-based systems, whose molecular recognition is sensitive to environmental conditions such as pH and ionic composition [25,82]. Beyond these effects on molecular function, the environmental behavior of DNA may also become more difficult to predict across heterogeneous soil conditions [133,134]. DNA degradation and persistence in soils can vary substantially with environmental factors such as moisture, temperature, organic carbon content, and habitat characteristics [133]. Notably, these matrix-dependent effects are not necessarily detrimental to DNA stability. Adsorption onto soil colloids and mineral surfaces can protect DNA against nuclease-mediated degradation, illustrating that interactions with environmental matrices may impair molecular accessibility or function while simultaneously enhancing DNA persistence [132]. Therefore, understanding and controlling these matrix-specific interactions and environmental effects will be important for achieving long-term functionality and stability of DNA-based remediation systems in complex environmental matrices and ultimately extending their applicability beyond water and wastewater treatment.

Another important consideration is the environmental safety and sustainability of the complete DNA-based material system. Although DNA itself is generally regarded as an environmentally compatible material owing to its low toxicity and biodegradability [13], many DNA-based remediation platforms incorporate additional components, including metal or metal oxide nanoparticles, carbon nanomaterials, MOFs, catalysts, and polymeric supports, whose potential environmental risks should be considered independently of the environmental compatibility of DNA [13,135]. In particular, during the remediation process, the potential release, persistence, and ecotoxicity of these supporting components should be carefully evaluated to avoid introducing secondary environmental risks [135]. Following remediation, the recoverability and reusability of the complete material system should also be assessed to ensure that the remediation process does not generate additional environmental burdens. These considerations should be addressed before practical implementation so that DNA-based remediation systems can be evaluated not only for their pollutant removal performance but also for their overall environmental impact and sustainability.

Economic feasibility represents another critical consideration for practical environmental remediation applications. The relatively high production cost of synthetic nucleic acids is one of the limitations always discussed in various fields when applying nucleic acid-based platforms [5,51,136]. Compared with conventional adsorbents or catalytic materials, synthetic nucleic acids remain relatively expensive, particularly for large-scale environmental applications. Fortunately, rapid advances in enzymatic DNA synthesis, automated high-throughput oligonucleotide manufacturing, and scalable production technologies are steadily reducing production costs while improving synthesis efficiency [62,137]. In parallel, hybrid designs integrating DNA with functional materials not only overcome several intrinsic limitations of DNA-only systems, such as limited structural stability and high production costs, but also substantially improve the practicality and scalability of DNA-based technologies [51,62]. For example, DNA has been immobilized onto cellulose scaffolds or encapsulated within PES hollow microspheres to improve structural stability, reusability, and pollutant removal efficiency, while aptamer encapsulation within liposomes has been shown to preserve molecular recognition activity under harsh conditions [59,82,94]. Although these studies remain largely at the proof-of-concept stage, they demonstrate that rational material engineering can substantially improve the robustness and practical feasibility of DNA-based remediation systems. Collectively, these continued efforts suggest that the long-standing limitations of DNA-based applications in various fields, including those of DNA-based remediation technology such as structural stability, reusability, and production cost, are gradually shifting from fundamental barriers to engineering challenges that can be addressed through rational material and process design. As these practical challenges become increasingly manageable, we propose that the future development of DNA-based remediation should extend beyond continued efforts to improve platform robustness and broaden applicability across diverse environmental matrices, with greater emphasis on broadening the range of pollutants that programmable DNA systems can selectively recognize and eliminate, expanding the functional repertoire of DNA, and diversifying the materials platforms with which DNA can be integrated.

### 5.2. Expanding DNA Functionalities

Over the past two decades, advances in DNA nanotechnology have led to the development of a wide variety of dynamic and programmable molecular systems, including DNAzymes, strand displacement circuits, HCR, CHA, and logic-gated DNA nanodevices [46,47,138,139]. Many of these technologies have been extensively investigated in biosensing, molecular diagnostics, and biomedical applications. However, most of these sophisticated DNA functionalities have yet to be fully exploited in environmental remediation. Current DNA-based remediation strategies primarily rely on the intrinsic adsorption properties of DNA and the sequence-specific molecular recognition capability of aptamers. Although these approaches have demonstrated excellent performance in broad-spectrum pollutant adsorption and selective pollutant capture, they represent only a limited subset of DNA’s functional capabilities, suggesting substantial opportunities to further expand the functional diversity of DNA-based environmental remediation systems. For example, despite their enormous potential to replace passive pollutant capture with irreversible detoxification, only a limited number of studies have explored catalytic DNAzymes for environmental remediation. Beyond directly catalyzing oxidative degradation reactions, DNAzymes may also function as programmable molecular actuators capable of initiating remediation processes. Cleavage-based DNAzymes could be engineered to activate pollutant degradation pathways, trigger the controlled release of catalytic agents through target-induced disassembly of DNA nanostructures, or operate as molecular switches that autonomously regulate remediation processes in response to specific environmental contaminants. Similarly, aptamers may serve not only as molecular recognition elements for selective pollutant capture but also as programmable molecular switches capable of initiating downstream responses following target recognition. Such target-responsive conformational changes could be coupled with catalytic activation, material assembly, or controlled payload release, thereby enabling more intelligent and autonomous remediation systems. Likewise, enzyme-free amplification systems such as HCR and CHA can autonomously generate higher-order DNA architectures or amplify molecular responses upon target recognition. As discussed above, translating these amplified molecular responses into enhanced remediation performance will require experimental validation demonstrating that they effectively improve pollutant capture, sequestration, or degradation. Nevertheless, their programmable and target-responsive nature provides opportunities to couple molecular recognition with functional components responsible for pollutant sequestration or transformation, potentially enabling the development of adaptive and stimuli-responsive remediation systems.

Another intriguing direction may be the development of in situ self-assembling DNA hydrogels. Rather than introducing pre-formed DNA hydrogels into contaminated environments, localized hydrogel formation triggered by specific environmental contaminants could potentially concentrate functional DNA components at contamination hotspots while minimizing unnecessary material consumption. Such an approach may improve material utilization and provide a more adaptive and spatially controlled remediation strategy. However, unlike biosensing systems that operate in confined reaction volumes, environmental remediation typically occurs in open and continuously diluted environments, where maintaining the local concentrations required for efficient enzyme-free amplification remains a major challenge. Furthermore, the relatively slow kinetics of DNA self-assembly compared with contaminant transport may limit hydrogel formation before pollutants diffuse away from the reaction site. Future efforts may therefore focus on integrating enzyme-free amplification reactions with solid substrates, porous scaffolds, membranes, or other confined interfaces to spatially localize hydrogel growth while preserving high local reactant concentrations. Such strategies could bridge the gap between programmable DNA self-assembly and practical environmental remediation. Importantly, many of these emerging DNA functionalities do not necessarily require entirely new remediation materials. Instead, they may be readily integrated into the numerous DNA-based platforms that have already been developed for environmental remediation. Existing DNA-functionalized hydrogels, membranes, graphene composites, MNPs, and other hybrid materials have primarily exploited DNA as a passive adsorbent or molecular recognition element. Incorporating emerging functionalities, such as DNAzyme-mediated catalysis, enzyme-free amplification, molecular logic operations, or stimuli-responsive self-assembly, into these established platforms could provide a pathway toward adaptive remediation systems that integrate sequential contaminant recognition, activation, and detoxification. Collectively, these emerging concepts suggest that the future role of DNA in environmental remediation extends beyond passive pollutant capture to adaptive molecular systems designed to integrate contaminant recognition, molecular information processing, functional self-assembly, and selective capture or catalytic detoxification.

### 5.3. Engineering Advanced Remediation Platforms

Current DNA-based remediation platforms remain relatively simple in their structural design, typically consisting of DNA immobilized on conventional supporting materials such as hydrogels, membranes, MNPs, or porous microspheres. While these platforms have significantly improved the environmental stability and recyclability of DNA and combined the advantages of each constituent material, future advancements are expected to depend on the development of more sophisticated material platforms capable of maximizing the performance of existing DNA-based molecular recognition and catalytic systems and expanding new DNA functions. Owing to its programmable molecular recognition, dynamic structural responsiveness, and excellent compatibility with a wide range of functional materials, DNA has been extensively integrated with diverse nanomaterials and hybrid platforms for applications such as biosensing, bioanalysis, and environmental monitoring. Representative examples include MOFs, covalent organic frameworks (COFs), MXenes, and nanozymes, all of which have been extensively functionalized with aptamers for these purposes while also being widely investigated as highly efficient materials for environmental remediation in their own right. Despite this substantial overlap, the integration of these nanomaterials with DNA for remediation applications remains surprisingly limited, highlighting an important opportunity to translate well-established DNA–material hybrid systems from sensing into next-generation environmental remediation platforms.

Therefore, integrating DNA with high-potential functional materials that have already demonstrated exceptional performance in biosensing or environmental remediation but have rarely been combined for pollutant removal can be a very promising direction for development. Among these, porous crystalline frameworks, including MOFs and COFs, represent particularly attractive candidates [140,141]. Although both MOFs and COFs are highly porous crystalline materials, they differ fundamentally in the chemical bonds that construct their frameworks. MOFs consist of metal ions connected by organic molecules through coordinate bonds, enabling intrinsic catalytic activity in addition to pollutant adsorption, whereas COFs are constructed entirely through covalent organic linkages, generally providing higher chemical stability and structural robustness. Both materials have been extensively investigated as adsorbents, catalysts, catalyst supports, and separation media for environmental remediation due to their exceptionally high surface area, tunable pore structures, and versatile chemical functionality. In parallel, DNA-functionalized MOFs and COFs have been widely developed for biosensing, molecular diagnostics, environmental monitoring, and controlled drug delivery because their porous architectures provide ideal scaffolds for programmable nucleic acid assembly [37,65,142]. However, these studies have primarily demonstrated the feasibility of integrating programmable DNA functions with MOF and COF architectures in non-remediation applications, while their direct application to DNA-based environmental remediation remains extremely limited.

Similarly, another emerging class of materials with considerable potential is MXenes, a family of crystalline two-dimensional nanomaterials composed of transition metals and carbon and/or nitrogen. Owing to their high electrical conductivity, excellent mechanical strength, chemical stability, abundant surface functional groups, outstanding adsorption capability, and efficient photothermal conversion properties, MXenes have attracted increasing attention across a wide range of environmental and bioanalytical applications [143]. Benefiting from these excellent properties, MXene has recently attracted considerable attention as a versatile material for water remediation, catalytic remediation, and radionuclide capture, while DNA–MXene hybrid systems have been rapidly developed in the field of biosensing, where MXene provides efficient nucleic acid immobilization, excellent signal transduction, and enhanced electrochemical performance [144,145]. Nevertheless, similar to MOFs and COFs, these two research directions have remained largely independent, and their utilization in DNA-based environmental remediation has rarely been reported.

Nanozymes represent another representative class of functional materials that have independently matured in both environmental remediation and DNA-based biosensing. Whereas MOFs, COFs, and MXenes primarily serve as multifunctional platforms that integrate molecular recognition with adsorption and catalytic processes, nanozymes represent a distinct class of intrinsically catalytic nanomaterials designed to mimic the functions of natural enzymes. Nanozymes have emerged as promising alternatives to natural enzymes because of their enzyme-like catalytic activity, excellent physicochemical stability, low production cost, and outstanding reusability [146]. Owing to their excellent properties, nanozymes have been extensively investigated for pollutant degradation, AOP, and water remediation [89]. In parallel, DNA-functionalized nanozymes have been widely developed as highly sensitive biosensing platforms, where aptamers or DNAzymes provide target-specific molecular recognition while nanozymes serve as catalytic signal amplifiers [90,91,147]. Among the studies integrating programmable DNA with nanozymes, one particularly noteworthy concept is the coronazyme, which combines the advantages of DNAzymes and nanozymes [148]. In this system, catalytic radicals are generated on the Au nanoparticle surface and subsequently transferred to the surrounding DNA corona, where substrate binding and catalytic turnover occur, resulting in substantially enhanced catalytic efficiency and reaction selectivity. More recently, the coronazyme concept has been further expanded by demonstrating that catalytic activity and substrate specificity can be independently optimized through the decoupled design of the nanozyme core and DNA corona [149]. This advancement highlights the exceptional modularity and programmability of DNA integrating nanozyme systems, providing a versatile platform for the development of next-generation artificial enzymes. Nevertheless, coronazymes are still in the proof-of-concept stage, and, as with the examples described earlier, research integrating programmable DNA and nanoenzymes for selective environmental remediation is extremely limited.

Collectively, MOFs, COFs, MXenes, and nanozymes represent representative classes of functional materials that have independently matured as both environmental remediation materials and DNA-based sensing platforms. Despite their high potential and remarkable convergence, these advances have remained largely fragmented across different research communities rather than being integrated into unified DNA-based remediation systems. This disconnect suggests that additional design considerations are required when adapting DNA–material hybrid systems for environmental remediation. Whereas monitoring primarily requires efficient molecular recognition and signal generation and amplification, practical remediation additionally demands high adsorption capacity, ease of mass transfer, long-term environmental stability, and repeated regeneration. Despite these differences, environmental monitoring and environmental remediation share many fundamental material design principles. The material platforms developed for these applications commonly possess a high specific surface area, efficient DNA immobilization, structural robustness, and excellent compatibility with programmable molecular recognition, providing a solid foundation for their adaptation to environmental remediation. Therefore, rather than developing entirely new DNA-integrated remediation platforms, a promising strategy would be to redesign these well-established DNA-integrated sensing platforms to prioritize the functions required for remediation, including high adsorption capacity, efficient mass transport, catalytic turnover, and structural durability. Such a transition could provide the potential for the advancement of next-generation DNA-based remediation systems.

The future of DNA-based remediation lies not only in building better platforms but also in broadening the range of pollutants that programmable DNA systems can selectively recognize and eliminate. In particular, the selective remediation of biological contaminants is a promising yet under-researched area of DNA-based remediation. Although existing biological contaminant removal technologies, including chlorination and UV irradiation, are highly effective in reducing the overall microbial burden, they rarely distinguish between harmful and beneficial microorganisms, and often show limited capability for selectively eliminating specific pathogenic species or biomolecular contaminants. By contrast, DNA aptamers capable of recognizing pathogenic bacteria, viruses, and disease-associated proteins have already been extensively developed for biosensing, diagnostic, and therapeutic applications [25,127,128,150]. These already developed molecular recognition elements can provide the potential to extend DNA-based remediation beyond conventional chemical pollutants to highly selective biological decontamination. Although several proof-of-concept studies have demonstrated the feasibility of DNA-based remediation of biological contaminants, this research direction remains considerably less explored than remediation strategies targeting heavy metals, organic pollutants, pharmaceuticals, and radionuclides [69,122,123,124,125,126]. This limited development is likely not due to the lack of suitable molecular recognition elements but rather because many of the intrinsic limitations of DNA, including structural instability, susceptibility to degradation, limited reusability, and high production costs, have historically constrained the practical deployment of DNA-based biological remediation systems. Therefore, overcoming the intrinsic engineering limitations of DNA and expanding its functional applications should not be regarded as independent research directions but rather as complementary efforts that together will determine the future development of DNA-based environmental remediation.

## 6. Conclusions

DNA has evolved from traditional, simple genetic material into a highly programmable engineering platform capable of molecular recognition, dynamic structural transformation, and functional integration with a wide range of materials. Importantly, DNA and conventional remediation materials can form complementary hybrid systems in which the established adsorption, catalytic, and structural properties of conventional materials are combined with the programmability and molecular functionality of DNA. This complementary integration gives DNA-based systems considerable potential as versatile remediation platforms by enabling multiple material and molecular functions to be incorporated within a single system. Although DNA-based environmental remediation remains at an early stage of development, experimentally demonstrated applications have been reported across diverse pollutant classes, including heavy metals, organic pollutants, pharmaceuticals, radionuclides, and biological contaminants. These studies span from laboratory-scale proof-of-concept demonstrations to validation under environmentally relevant conditions, although the latter remains relatively limited.

Major barriers to practical implementation remain, including matrix-dependent variations in molecular performance, structural and functional stability under complex environmental conditions, production costs and scalability, long-term durability, material recovery and reuse, and the environmental safety of complete hybrid material systems. Recent advances in hybrid materials engineering, DNA synthesis and manufacturing, and immobilization strategies suggest that some of these limitations are increasingly becoming addressable engineering challenges rather than inherent constraints of DNA-based remediation systems. However, addressing these barriers will require further systematic investigation and validation under environmentally relevant conditions, together with continued improvements in material design, manufacturing, and process engineering.

Beyond overcoming these practical and engineering barriers, a second important direction for future development is the integration and expansion of the diverse yet currently fragmented DNA functionalities, material platforms, and remediation targets explored across different studies. Before emerging DNA-programmed functions are translated into environmental remediation, however, their suitability and actual benefits for remediation should first be experimentally validated, particularly for functions such as enzyme-free amplification, molecular logic operations, and stimuli-responsive self-assembly that have been primarily explored in other fields. These two directions—overcoming practical and engineering barriers and integrating and expanding currently fragmented functionalities, material platforms, and remediation targets—should not be regarded as independent objectives but rather as complementary efforts that should progress in parallel. If progress along these two complementary directions can be achieved, DNA-based remediation may evolve into a more versatile platform capable of supporting highly selective, adaptive, and environmentally sustainable remediation strategies.

## Figures and Tables

**Table 1:** Summary of representative DNA-based heavy metal remediation studies.

Binding Mechanism	Target Heavy Metal Ions	Function	References
Electrostatic adsorption	Hg^2+^, Cu^2+^, Pb^2+^, Cd^2+^, etc.	Broad-spectrum adsorption	[57,63,94]
Metal-mediated base pairing	Hg^2+^	Selective capture	[56,68,70,80,83,95]
Structure-dependent recognition	Mn^2+^		[96]
	As(III), As(V)		[97]
	Pb^2+^		[98]
	Co^2+^	Material recovery	[99]
	UO_2_^2+^		[100,101,102,103]

**Table 3:** Summary of representative DNA-based biological contaminant remediation studies.

Target Biological Contaminants	The Role of DNA	Remediation Mechanism	References
Hepatitis C virus (HCV)	Aptamer	Selective capture and removal	[122]
Hepatitis B virus surface antigen (HBsAg)			[123]
*Listeria monocytogenes*		Targeted antimicrobial delivery	[69]
*Escherichia coli* (*E. coli*)			[124]
		Selective recognition and photocatalytic therapy	[125]
Methicillin-resistant *Staphylococcus aureus* (MRSA)		Magnetic separation and photothermal therapy	[126]

## Data Availability

No new data were created or analyzed in this study. Data sharing is not applicable to this article.
